# Tau topography subtypes account for clinical heterogeneity and longitudinal trajectories in early-onset Alzheimer's disease

**DOI:** 10.1093/braincomms/fcag176

**Published:** 2026-05-18

**Authors:** Marlene Lin, Konstantinos Chiotis, Piyush Maiti, Jiaxiuxiu Zhang, Ganna Blazhenets, Salma Rocha, Ranjani Shankar, Alinda Amuiri, Dustin B Hammers, Ani Eloyan, Kala Kirby, Robert A Koeppe, Paul Aisen, Laurel Beckett, Walter A Kukull, Arthur W Toga, Alireza Atri, David G Clark, Gregory S Day, Ranjan Duara, Neill R Graff-Radford, Ian Grant, Lawrence S Honig, Erik C B Johnson, David T Jones, Joseph C Masdeu, Mario F Mendez, Erik Musiek, Chiadi U Onyike, Meghan Riddle, Emily Rogalski, Stephen Salloway, Sharon J Sha, Raymond Scott Turner, Thomas S Wingo, David A Wolk, Kyle B Womack, Kelly Nicole Holohan Nudelman, Alexandra Touroutoglou, Clifford R Jack, Prashanthi Vemuri, Jacob W Vogel, Suzanne M Dufault, Thomas J Hoffmann, Maria C Carrillo, Bradford C Dickerson, Liana G Apostolova, Gil D Rabinovici, Renaud La Joie

**Affiliations:** Edward and Pearl Fein Memory and Aging Center, Weill Institute for Neurosciences, Department of Neurology, University of California San Francisco, San Francisco, CA, USA; Edward and Pearl Fein Memory and Aging Center, Weill Institute for Neurosciences, Department of Neurology, University of California San Francisco, San Francisco, CA, USA; Center for Alzheimer Research, Division of Clinical Geriatrics, Department of Neurobiology, Care Sciences and Society, Karolinska Institutet, Stockholm, Sweden; Department of Cognitive Disorders, Karolinska University Hospital, Stockholm, Sweden; Edward and Pearl Fein Memory and Aging Center, Weill Institute for Neurosciences, Department of Neurology, University of California San Francisco, San Francisco, CA, USA; Edward and Pearl Fein Memory and Aging Center, Weill Institute for Neurosciences, Department of Neurology, University of California San Francisco, San Francisco, CA, USA; Edward and Pearl Fein Memory and Aging Center, Weill Institute for Neurosciences, Department of Neurology, University of California San Francisco, San Francisco, CA, USA; Edward and Pearl Fein Memory and Aging Center, Weill Institute for Neurosciences, Department of Neurology, University of California San Francisco, San Francisco, CA, USA; Edward and Pearl Fein Memory and Aging Center, Weill Institute for Neurosciences, Department of Neurology, University of California San Francisco, San Francisco, CA, USA; Edward and Pearl Fein Memory and Aging Center, Weill Institute for Neurosciences, Department of Neurology, University of California San Francisco, San Francisco, CA, USA; Department of Neurology, Indiana University School of Medicine, Indianapolis, IN, USA; Department of Biostatistics, Brown University, Providence, RI, USA; Department of Neurology, Indiana University School of Medicine, Indianapolis, IN, USA; Department of Radiology, University of Michigan, Ann Arbor, MI, USA; Alzheimer’s Therapeutic Research Institute, University of Southern California, San Diego, CA, USA; Department of Public Health Sciences, University of California Davis, Davis, CA, USA; Department of Neurology, Washington University School of Medicine in St. Louis, St. Louis, MO, USA; Laboratory of Neuro Imaging, USC Stevens Neuroimaging and Informatics Institute, Keck School of Medicine of USC, Los Angeles, CA, USA; Banner Sun Health Research Institute, Sun City, AZ, USA; Department of Neurology, Indiana University School of Medicine, Indianapolis, IN, USA; Department of Neurology, Mayo Clinic, Jacksonville, FL, USA; Wien Center for Alzheimer’s Disease and Memory Disorders, Mount Sinai Medical Center, Miami Beach, FL, USA; Department of Neurology, Mayo Clinic, Jacksonville, FL, USA; Department of Psychiatry and Behavioral Sciences, Mesulam Center for Cognitive Neurology and Alzheimer’s Disease, Feinberg School of Medicine, Northwestern University, Chicago, IL, USA; Taub Institute and Department of Neurology, Columbia University Irving Medical Center, NewYork, NY, USA; Department of Neurology, Emory University School of Medicine, Atlanta, GA, USA; Department of Radiology, Mayo Clinic, Rochester, MN, USA; Nantz National Alzheimer Center, Houston Methodist and Weill Cornell Medicine, Houston, TX, USA; Department of Neurology, David Geffen School of Medicine at UCLA, Los Angeles, CA, USA; Department of Neurology, Washington University School of Medicine in St. Louis, St. Louis, MO, USA; Department of Psychiatry and Behavioral Sciences, Johns Hopkins University School of Medicine, Baltimore, MD, USA; Department of Psychiatry, Alpert Medical School, Brown University, Providence, RI, USA; Healthy Aging & Alzheimer’s Research Care Center, Department of Neurology, University of Chicago, Chicago, IL, USA; Department of Psychiatry, Alpert Medical School, Brown University, Providence, RI, USA; Department of Neurology & Neurological Sciences, Stanford University, Palo Alto, CA, USA; Department of Neurology, Georgetown University, Washington, DC, USA; Department of Neurology, University of California, Davis, Sacramento, CA, USA; Department of Neurology, Perelman School of Medicine, University of Pennsylvania, Philadelphia, PA, USA; Department of Neurology, Washington University School of Medicine in St. Louis, St. Louis, MO, USA; Department of Medical and Molecular Genetics, Indiana University School of Medicine, Indianapolis, IN, USA; Department of Neurology, Massachusetts General Hospital and Harvard Medical School, Boston, MA, USA; Department of Radiology, Mayo Clinic, Rochester, MN, USA; Department of Radiology, Mayo Clinic, Rochester, MN, USA; Department of Clinical Sciences Malmö, Faculty of Medicine, SciLifeLab, Lund University, Lund, Sweden; Department of Epidemiology and Biostatistics, University of California San Francisco, San Francisco, CA, USA; Department of Epidemiology and Biostatistics, University of California San Francisco, San Francisco, CA, USA; Medical & Scientific Relations Division, Alzheimer’s Association, Chicago, IL, USA; Department of Neurology, Massachusetts General Hospital and Harvard Medical School, Boston, MA, USA; Department of Neurology, Indiana University School of Medicine, Indianapolis, IN, USA; Edward and Pearl Fein Memory and Aging Center, Weill Institute for Neurosciences, Department of Neurology, University of California San Francisco, San Francisco, CA, USA; Department of Radiology and Biomedical Imaging, University of California San Francisco, San Francisco, CA, USA; Edward and Pearl Fein Memory and Aging Center, Weill Institute for Neurosciences, Department of Neurology, University of California San Francisco, San Francisco, CA, USA

**Keywords:** neuroimaging biomarkers, disease heterogeneity, atypical Alzheimer's disease, data-driven clustering, voxel-wise modelling

## Abstract

The growing availability of large-scale biomarker datasets has allowed data-driven methods to characterize Alzheimer’s disease biological heterogeneity. However, most prior studies have focused on cohorts of late-onset amnestic cases, leaving early-onset Alzheimer’s disease underexplored. We aimed to characterize tau-PET-based subtypes through a robust data-driven approach in the Longitudinal Early-Onset Alzheimer’s Disease Study.

Baseline [^18^F]Flortaucipir PET scans from 365 amyloid-PET-positive participants with sporadic early-onset Alzheimer’s disease were quantified in the left and right medial temporal, lateral temporal, occipital, parietal, and frontal cortices. Tau PET values were z-scored against 85 amyloid-PET-negative cognitively normal age-matched participants and fitted into Subtype and Stage Inference (SuStaIn)—an unsupervised clustering algorithm that simultaneously models subtypes and progression from cross-sectional data. The derived subtypes were subsequently characterized by baseline and longitudinal clinical, cognitive, MRI, tau and amyloid PET features. We identified three tau-PET-based subtypes: on average, Subtype 1/Typical (*n* = 144, 40%) showed a predominant bilateral temporoparietal pattern typical of Alzheimer’s disease. Subtype 2/Left temporal (*n* = 111, 31%) showed predominant left temporal binding. Subtype 3/Posterior (*n* = 104, 29%) showed early and permeating occipitoparietal involvement. Subtypes did not differ in demographics or global amyloid burden, but were relatively more enriched for specific clinical presentations: S1/Typical for amnestic presentations, S2/Left Temporal for primary progressive aphasia, and S3/Posterior for posterior cortical atrophy. Baseline tau PET subtypes aligned with cortical atrophy patterns and domain-specific cognitive impairment. When follow-up tau PET scans were fitted to SuStaIn trained on baseline data, 85.6% (*n* = 172/201) of participants retained the same subtype classification, indicating subtype temporal stability, and progressed within subtypes by 0.56 ± 0.70 SuStaIn stage/year. Longitudinal voxel-wise linear mixed-effects modelling revealed tau accumulation patterns for each subtype in regions relatively spared at baseline: occipital lobe accumulation predominated in S1/Typical, bilateral frontal and right temporal in S2/Left Temporal, and bilateral frontotemporal lobes in S3/Posterior. All subtypes showed longitudinal increases in Clinical Dementia Rating-Sum of Boxes, but with slower worsening in S3/Posterior compared with the other subtypes. Our findings reveal robust subtypes in sporadic early-onset Alzheimer’s disease characterized by distinct spatiotemporal tau patterns that parallel differences in clinical presentations and trajectories of neurodegeneration. These subtypes extend beyond traditional clinical syndromes and support a more nuanced framework for individualized prognosis and care. Incorporating tau PET subtyping into clinical trial design could enable more targeted therapeutic approaches for this younger population.

## Teaser

Applying data-driven modelling to tau PET data in sporadic early-onset Alzheimer disease (age < 65), Lin *et al*. identified three longitudinally stable subtypes characterized by distinct spatiotemporal patterns. These tau PET subtypes differed in clinical presentation, cognitive trajectories, and neurodegeneration, highlighting biologically meaningful heterogeneity with relevance for disease monitoring and intervention strategies.

## Graphical Abstract

**Graphical Abstract:**
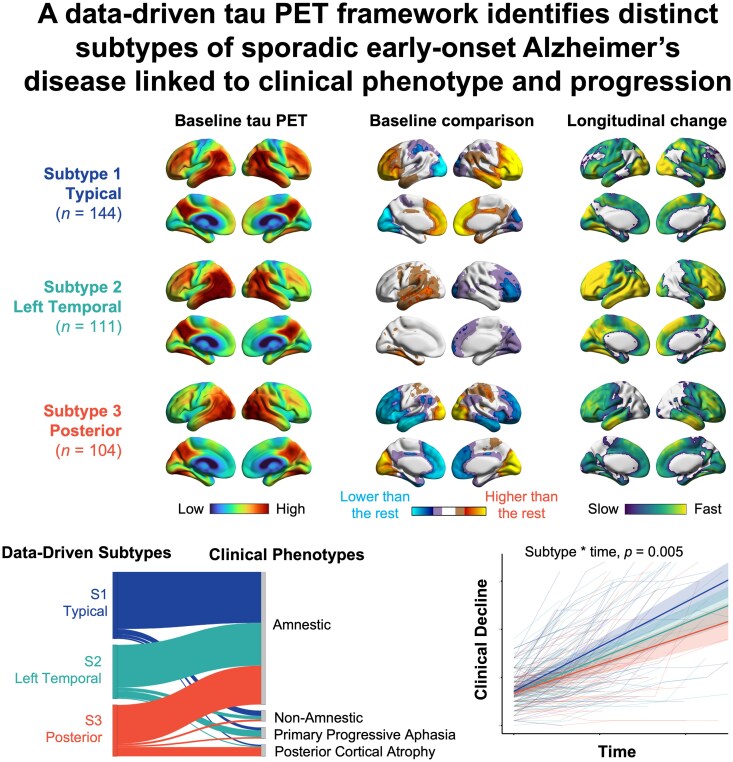

## Introduction

Alzheimer’s disease is characterized by the aggregation of amyloid-β (Aβ) into extracellular plaques and hyperphosphorylated tau into intraneuronal tangles.^1^ Neuropathological investigations have delineated the stereotypical progression of tau tangles in limbic and cerebral cortex, initiating in the medial temporal lobe and extending to inferior and lateral temporal cortex, and other neocortical regions as cognitive symptoms develop and the disease advances.^2,3^ However, much of this foundational knowledge is derived from postmortem examinations of late-stage, late-onset cases, frequently from selective clinical cohorts, and often with limited anatomical sampling. These constraints restrict our understanding of disease progression and hamper examination of inter-individual variability.

Alzheimer’s disease is clinically heterogeneous, with patients differing in clinical phenotype (e.g. memory-predominant or language-predominant presentations), rate of progression, and age at symptom onset.^4-7^ Recent research has leveraged fluid- and imaging-based biomarkers to better understand the biological underpinnings of this variability. Among biomarkers, tau-related measures show the strongest associations with symptom severity and cognitive decline, making them a critical target for elucidating links between biological variation and clinical outcomes.^8^ The advent of tau PET imaging has enabled in vivo visualization of tau pathology load and distribution, complementing knowledge from autopsy-based studies and enabling investigation of large cohorts with longitudinal follow-up.

In parallel, data-driven modelling approaches—including clustering methods—have been increasingly used to identify biologically meaningful subtypes based on high-dimensional biomarker data, independent of clinical classifications. Applied to tau PET, these methods could reveal distinct patterns of tau propagation. Notably, a seminal study by Vogel *et al*.^9^ gathered multiple cohorts of patients with tau PET and applied SuStaIn, a probabilistic unsupervised machine learning model that infers distinct disease subtypes and their temporal progressions.^10^ Using this method, authors identified four spatiotemporal tau patterns that differed in demographical, clinical, and genetic features. However, this previous paper, similar to most prior studies, mainly included older (≥65 years of age) patients with typical amnestic-predominant presentations of Alzheimer’s disease and often lacked sufficient longitudinal analyses to determine whether cross-sectional subtypes reflect meaningful differences in disease progression outcomes such as brain atrophy and cognitive decline.^11-15^

Early-onset Alzheimer’s disease (EOAD), defined by symptom onset before age 65, accounts for approximately 5–10% of cases with Alzheimer’s disease; 90–95% of individuals with EOAD are sporadic, they do not carry a known disease-causing mutation.^6,16^ Compared with late-onset, early-onset patients typically exhibit greater amyloid and tau burden, more severe cognitive impairment, more frequent non-amnestic phenotypes, and fewer age-related comorbidities.^17-22^ These characteristics make the early-onset cohort a useful model for studying core disease mechanisms, including tau-related heterogeneity, in the relative absence of age-associated mixed pathologies. Nonetheless, studies specifically investigating heterogeneity within early-onset cohorts remain limited.^23^

To examine heterogeneity in tau deposition among individuals with EOAD from the ongoing Longitudinal Early-Onset Alzheimer’s Disease Study (LEADS),^24^ SuStaIn was used to identify tau-PET-based subtypes in EOAD and reconstruct subtype-specific sequences of regional tau accumulation. We then evaluated how these subtypes differ in baseline and comprehensive longitudinal cognitive profiles. We further validated the progression patterns using longitudinal tau PET data from the same individuals and compared the subtypes with respect to upstream Aβ accumulation and downstream neurodegeneration over time.

## Materials and methods

### LEADS study design

The Longitudinal Early-Onset Alzheimer’s Disease Study (LEADS) is a prospective, multisite observational clinical and biomarker study (NCT03507257) that began in 2018. LEADS aims to characterize the pathophysiological mechanisms, heterogeneity, and heritability of sporadic EOAD, and identify optimal outcome measures for trials in this population.^24^ The study enrols cognitively-impaired (CI) participants aged 40–64 at the time of consent, meeting the National Institute on Aging and the Alzheimer’s Association criteria for mild cognitive impairment (MCI) or mild dementia due to Alzheimer’s disease with a Clinical Dementia Rating (CDR) ≤ 1, and cognitively normal (CN) age-matched controls with a Mini-Mental State Examination (MMSE) score ≥ 24 and a global CDR of 0.^25^ LEADS does not restrict participation to individuals with amnestic presentations, thereby allowing for characterization of a broader phenotypic spectrum of sporadic EOAD.^19^ Participants with known pathogenic variants in the APP, PSEN1, PSEN2, MAPT, GRN, or C9ORF72 genes are ineligible. All enrolled participants undergo genetic screening to further ensure exclusions of any carriers of these mutations.^26^

At baseline, CI participants are classified into the EOAD and early-onset non-Alzheimer’s disease (EOnonAD) group based on centrally-read [^18^F]Florbetaben PET scans supported by quantitative analysis: those with a positive amyloid PET scan are classified as EOAD, while those with a negative scan are categorized as EOnonAD.^21,23^

LEADS study procedures followed the Good Clinical Practice guidelines and applicable federal and state regulations for the protection of human research participants. The study uses Indiana University as the single Institutional Review Board of record. Site-specific regulatory documents, including informed consent forms and Health Insurance Portability and Accountability Act authorizations, are adapted from IU-approved templates to comply with local requirements. Written informed consent is obtained from all participants or their legally authorized representatives in accordance with the Declaration of Helsinki and IRB-approved procedures.

### Sample of interest

Although this project aims to identify EOAD subtypes, baseline tau PET data from all participants, regardless of clinical status and amyloid PET positivity, were included for SuStaIn input preparation. However, only the EOAD group was used to run the SuStaIn algorithm (see ‘Input’). As per LEADS protocol, participants with EOAD undergo clinical and neuroimaging assessments at baseline, 12, 24, and 36 months, with an additional clinical visit after 48 months. This project included data downloaded from the study’s repositories as of February 2025 for participants enrolled before July 2024.

### Data acquisition and pre-processing

#### PET and MRI

MRI and PET acquisitions and pre-processing were detailed previously.^21,23,24^ MRI scans were acquired across all sites using 3T scanners following a standardized protocol harmonized with Alzheimer's Disease Neuroimaging Initiative 3 (ADNI3). T1-weighted MRI were processed using Freesurfer 7.1 to obtain a cortical and subcortical parcellation in native space.

Amyloid PET was acquired 90–110 min after injecting ∼8 mCi of [^18^F]Florbetaben (four 5-min frames). Tau PET was acquired 75–105 min after injecting ∼10 mCi of [^18^F]Flortaucipir (six 5-min frames). For each PET session, frames were realigned, averaged and smoothed to harmonize resolution to an estimated 6 mm full width at half maximum (FWHM). PET images were then co-registered to T1-weighted MRI scans acquired within a year for region-based quantification using Freesurfer parcellation. Standardized uptake value ratio (SUVR) values were calculated using the whole cerebellum as the reference region for [^18^F]Florbetaben and inferior cerebellar cortex for [^18^F]Flortaucipir. All PET analyses were performed on non–partial volume corrected data. Scans failing quality control standards were excluded from further analyses. A composite neocortical [^18^F]Florbetaben SUVR was derived and converted to Centiloid units using a published equation.^27^ Two metrics were extracted from the [^18^F]Flortaucipir SUVR images: a temporal meta-ROI, and a volume-weighted average of all cortical ROIs, to measure global cortical tau burden.^23^

To run voxel-wise analyses, T1-weighted MRIs were segmented, warped to the Montreal Neurological Institute Space, and modulated using Statistical Parametric Mapping 12 (SPM12). Total Intracranial Volume (TIV) was also derived from SPM12. A Gaussian kernel of 8 mm FWHM was applied to the modulated, warped, grey matter images. [^18^F]Florbetaben and [^18^F]Flortaucipir images were warped to MNI space using the deformation matrix derived from the MRI procedure.

#### Clinical and cognitive data

Participants’ clinical presentations were classified through multidisciplinary consensus conferences along multiple dimensions based on previously published criteria, including severity (MCI^28^ or dementia^25^) and phenotype (amnestic-predominant, single or multiple domain; primary progressive aphasia [PPA];^29^ posterior cortical atrophy [PCA];^30^ or other non-amnestic, single or multiple domain presentations). Apolipoprotein E (ApoE) genotyping of rs429358 and rs7412 was performed on blood-derived DNA, and carrier status was defined by the presence of both ε4 alleles.

Cognitive assessments were conducted at baseline and follow-up visits using a combination of standardized instruments (Supplementary Method 1).

### Subtype and stage inference

#### Algorithm

SuStaIn is an unsupervised, probabilistic machine learning algorithm (Supplementary Fig. 1A) that simultaneously infers disease subtypes and their corresponding biomarker trajectories using cross-sectional data. The linear version of SuStaIn models disease progression as a sequence of discrete events, or monotonic stages, each corresponding to a regional biomarker reaching predefined severity thresholds. Within each region, progression is represented as a piecewise linear function across these thresholds. To identify distinct subtypes, SuStaIn performs divisive hierarchical clustering and uses the expectation-maximization algorithm to jointly optimize subtype-specific event sequences and the assignment of individuals to subtypes concurrently. A full mathematical formulation is provided in Young *et al*.^31^

#### Input

The SuStaIn algorithm relies on the definition of a priori severity thresholds to represent discrete events along measures of interest. For our [^18^F]Flortaucipir PET data, these measures of interest were average SUVR values extracted from 10 unilateral lobar composite regions-of-interest (ROIs) derived from the Freesurfer parcellation (Supplementary Fig. 1B, Supplementary Table 1), including left and right frontal, parietal, lateral temporal, occipital, medial-temporal, as defined by Vogel *et al*.^9^ For each ROI, two data-driven thresholds were selected based on 2-component Gaussian Mixture Modelling (2-GMM) fitted across CN and EOAD participants, yielding a 20-event model that aligned with empirical recommendations of at least 10–20 observations per event, ensuring adequate power for subtype and stage estimation.^9^ Details of SuStaIn input preparation and model implementation are provided in Supplementary Method 2, Supplementary Figs 2–4, and Supplementary Table 2.

#### Model fitting and output

SuStaIn was applied to baseline [^18^F]Flortaucipir PET data from 365 participants with EOAD, using z-scored SUVR values from 10 lobar ROIs and the ROI-specific severity thresholds as input features. CN participants were used only for z-scoring and were excluded from SuStaIn model fitting and all subsequent analyses. The model was implemented with the package-suggested configurations (Supplementary Table 3). Each participant’s baseline scan was assigned to the subtype with the highest likelihood, and then to the stage with the highest probability within that subtype. Poorly fitted cases were defined as scans with probabilities belonging to any subtypes below 50%, and the corresponding participants were excluded from all post-clustering analysis. To assess the robustness of our exclusion criteria for participants with poor model fit, we repeated the primary analyses using more lenient (0%) and stricter (80%) subtype probability thresholds.

The optimal number of subtypes was determined through 5-fold cross-validation based on criteria including cross-validation information criteria and test-set log-likelihood. Changes in individual subtype assignment and comparisons among the derived subtypes were visualized as the number of subtypes increased, to assess whether additional subtypes contributed meaningful distinctions. These complementary outputs collectively informed the final model selection.

### Statistical analysis

After fitting SuStaIn to baseline [^18^F]Flortaucipir PET data from participants with EOAD, we aimed to characterize and compare the resulting subtypes in terms of clinical, cognitive, and neuroimaging features, both at baseline and over time.

#### Baseline subtype characterization

To visualize the spatial distribution and within-subtype progression of tau burden at baseline, we generated average [^18^F]Flortaucipir SUVR images across SuStaIn subtypes and stages. [^18^F]Flortaucipir PET, [^18^F]Florbetaben PET and T1-weighted MRI images were contrasted between each subtype and the rest to assess regional variations in tau burden, amyloid burden, and cortical atrophy. All voxel-wise comparisons were adjusted for baseline covariates, including age, sex, SuStaIn stage, and education, which has no missingness. For [^18^F]Flortaucipir PET and MRI comparisons, baseline Centiloid values were also adjusted for, and MRI comparison further adjusted for TIV. Subtype-versus-rest voxel-wise comparisons were conducted using SPM12, applying double stringency thresholds (uncorrected *P* < 0.001; adjusted for family-wise error [FWE] using Random Field Theory, *P* < 0.05) to highlight regions with robust between-group differences.^8,32^

Baseline demographic, clinical, and cognitive variables were compared across subtypes using analysis of variance (ANOVA) for continuous variables and chi-squared tests for categorical variables using the Python tableone package (v0.9.1).^33^ Baseline cognitive performance was further analysed using linear regression models to assess the association between SuStaIn stage and individual test scores. Models were implemented in R (v4.3.1), included subtype-by-stage interactions, and were adjusted for baseline age, sex, education, and Centiloid.

#### Subtype stability and staging consistency over time

[^18^F]Flortaucipir PET scans acquired at follow-up visits were subtyped and staged using the model trained on baseline data, with all assignments performed blind to participant identity and follow-up timepoints to prevent information leakage. To evaluate the longitudinal robustness of SuStaIn classification, we first assessed stability, defined as a participant retaining the same subtype assignment across timepoints and quantified through a confusion matrix and Cohen’s kappa (*κ*) coefficient.^34^ Characteristics of participants who changed subtypes were also examined.

We next assessed SuStaIn stage progression for individuals who remained in the same subtype, to verify that later follow-up scans were assigned to the same or a more advanced stage, reflecting progression in the estimated trajectory over time. One-sample *t*-tests were conducted within each subtype to determine whether stage increased significantly, while ANOVA was used to examine differences in stage progression rates across subtypes.

#### Longitudinal changes by baseline subtypes

Longitudinal changes in tau, amyloid, atrophy, and cognition by baseline subtype were modelled using linear mixed-effects (LME) models, with time, baseline subtype, and their interaction as primary predictors, and participant-specific random effects to capture individual variability. Models were adjusted for demographic and imaging covariates (Supplementary Method 3).

## Results

The final sample included 365 amyloid-PET-positive participants with EOAD (Table 1), and 85 amyloid-PET-negative CN controls (female: *n* = 54/85, 64%; age: 56.7 ± 5.9). Of the full dataset (*n* = 450), 389 participants (86%) identified as White, 35 (8%) as Black/African American, and 15 (3%) as Asian. 21 participants (5%) identified as Hispanic. CN participant characteristics are documented in Supplementary Table 4.

**Table 1:** SuStaIn subtype baseline demographic, clinical, and cognitive characteristics

Variables	Missing	Total (*n* = 359)	S1/Typical (*n* = 144)	S2/Left Temporal (*n* = 111)	S3/Posterior (*n* = 104)	*P*-Value
**Demographics**						
Years of Education	0	15.6 (2.4)	15.6 (2.5)	15.6 (2.4)	15.8 (2.4)	0.66
Age	0	59.1 (4.0)	58.9 (4.1)	58.9 (3.9)	59.6 (3.9)	0.31
Sex—Female	0	198 (55.2)	78 (54.2)	63 (56.8)	57 (54.8)	0.92
ApoE4 Carrier	9	195 (54.3)	71 (49.3)	59 (53.2)	65 (62.5)	0.36
**Neuroimaging**						
[^18^F]Florbetaben, Centiloid	0	103.1 (27.9)	104.0 (29.5)	103.3 (24.5)	101.6 (29.0)	0.80
[^18^F]Flortaucipir SUVR (all cortical ROIs)	0	1.9 (0.4)	2.0 (0.5)	2.0 (0.3)	1.8 (0.4)	**0**.**04**
[^18^F]Flortaucipir SUVR (temporal Meta ROI)	0	2.2 (0.5)	2.3 (0.5)	2.3 (0.4)	2.1 (0.5)	**<0**.**001**
SuStaIn Stage	0	12.1 (3.7)	12.1 (4.2)	12.6 (2.6)	11.8 (3.9)	0.30
**Clinical**						
MoCA	28	15.5 (6.2)	15.3 (6.4)	14.5 (6.1)	16.9 (5.6)	**0**.**02**
CDR-SB^+^	3	3.9 (2.0)	4.1 (2.3)	3.7 (1.8)	3.8 (1.8)	0.22
MMSE	9	21.3 (5.4)	21.1 (5.7)	20.6 (5.5)	22.2 (4.6)	0.07
Diagnosis—Dementia	1	267 (74.4)	108 (75.0)	84 (75.7)	75 (72.1)	0.61
Clinical Phenotype—Amnestic	0	290 (80.8)	124 (86.1)	88 (79.3)	78 (75.0)	**<0**.**001**
**Detailed Neuropsychological Battery**						
Benson Figure Copy	35	11.2 (5.3)	12.3 (5.0)	11.3 (5.0)	9.6 (5.7)	**0**.**001**
Line Orientation^+^	153	8.4 (4.4)	7.9 (4.3)	7.9 (4.1)	9.6 (4.7)	**0**.**04**
Line Length^+^	115	6.1 (4.7)	5.5 (4.2)	5.6 (3.9)	7.4 (5.7)	**0**.**02**
Trail Making Test Part A^+^	53	73.4 (46.5)	64.8 (42.9)	75.8 (46.7)	82.4 (49.2)	**0**.**02**
Digit Span Forward	22	5.9 (2.5)	6.1 (2.7)	5.6 (2.4)	6.1 (2.4)	0.21
Semantic Fluency (Vegetable + Animal)	25	19.0 (8.6)	19.1 (9.3)	16.3 (7.4)	21.8 (8.0)	**<0**.**001**
Multi-Lingual Naming Test	29	25.9 (5.8)	26.0 (6.1)	24.2 (6.7)	27.7 (3.1)	0.05
Trail Making Test Part B^+^	165	194.4 (93.5)	180.2 (92.5)	218.7 (89.4)	185.5 (95.2)	**0**.**04**
Digit Span Backward	25	4.0 (2.3)	4.0 (2.4)	3.6 (2.0)	4.2 (2.4)	0.14
Phonemic Verbal Fluency (L + F)	32	19.2 (10.7)	19.1 (11.8)	17.1 (10.2)	21.5 (9.4)	**0**.**02**
Digit Symbol Substitution Test	61	24.0 (16.4)	25.6 (16.8)	21.7 (15.2)	24.5 (17.2)	0.21
Match	86	22.9 (16.4)	23.8 (16.5)	21.5 (15.5)	23.2 (17.2)	0.61
Flanker	118	3.7 (3.2)	3.9 (3.2)	3.6 (3.2)	3.4 (3.3)	0.66
RAVLT Delayed Recall	25	1.8 (2.8)	1.6 (2.5)	2.0 (3.2)	1.9 (2.6)	0.59
Craft Story 21 Delayed Recall	35	4.1 (4.4)	4.2 (4.7)	3.2 (3.2)	4.9 (4.9)	**0**.**03**
Benson Figure Delayed Recall	52	3.3 (3.6)	3.7 (3.7)	3.4 (3.8)	2.8 (3.1)	0.25

Values are reported as mean (SD) for continuous variables, *n* (%) for categorical variables. *P*-values reflect omnibus tests comparing the three subtypes, ANOVA for continuous variables, chi-squared tests for categorical variables. *P*-values < 0.05 are in bold. Plus sign (^+^): higher score on this cognitive test indicates worse performance. Otherwise, lower score indicates worse performance.

### Model fit and subtype number choice

A three-subtype solution was selected based on the elbow point observed in cross-validation information criteria and test set log-likelihood across models with one to six subtypes, representing an optimal balance between model fit and parsimony (Supplementary Fig. 1C). In line with the quantitative results, the alluvial plot (Supplementary Fig. 1D) illustrates the redistribution of participants when classified into up to six subtypes, with S1 and S3 from the three-subtype solution remaining relatively stable across solutions, while S2 showed more frequent splits. The probability of participants’ assignment to each of the three subtypes is shown in Supplementary Fig. 1E, with most (*n* = 359/365, 98%, 95% CI: 97–100%) classified to a distinct subtype with high certainty. The participants with poor model fit (*n* = 6/365, 2%, 95% CI: 0–3%), who showed less than 50% probability of belonging to any subtype (i.e. within the inner triangle), were excluded from subsequent analyses. Subtype average [^18^F]Flortaucipir PET SUVR images and subtype-versus-rest-comparisons for different solutions are provided in Supplementary Figs 5 and 6.

Sensitivity analyses using alternative thresholds for exclusion of participants with poor model fit (0% and 80%) confirmed that the resulting baseline subtype profiles were robust (Supplementary Figs 7 and 8 and Supplementary Tables 5 and 6). Comparisons between participants excluded due to model fit and the participants included in baseline analyses were shown in Supplementary Table 7; briefly, excluded patients were older, less clinically impaired, and had lower amyloid and tau PET burden than patients assigned to a subtype. The stage distribution for the three-subtype solution is provided in Supplementary Fig. 9 and Supplementary Table 8. Each subtype demonstrated a largely stepwise progression pattern, with participants fulfilling the events up to their assigned SuStaIn stages. On average, participants deviated by 2.61 (95% CI: 2.39–2.84), 3.08 (95% CI: 2.78–3.38), and 2.67 (95% CI: 2.37–2.97) events from their assigned subtype’s sequence of 20 events for S1, S2, and S3, respectively, with significant differences across subtypes (*F*(2, 362) = 3.4; *P* = 0.03). Most deviations occurred in medial temporal regions. Event heatmaps and region-wise imperfection plots illustrating these patterns are provided in Supplementary Fig. 10.

### Subtypes exhibit distinct tau PET spatiotemporal patterns

**Figure 1:**
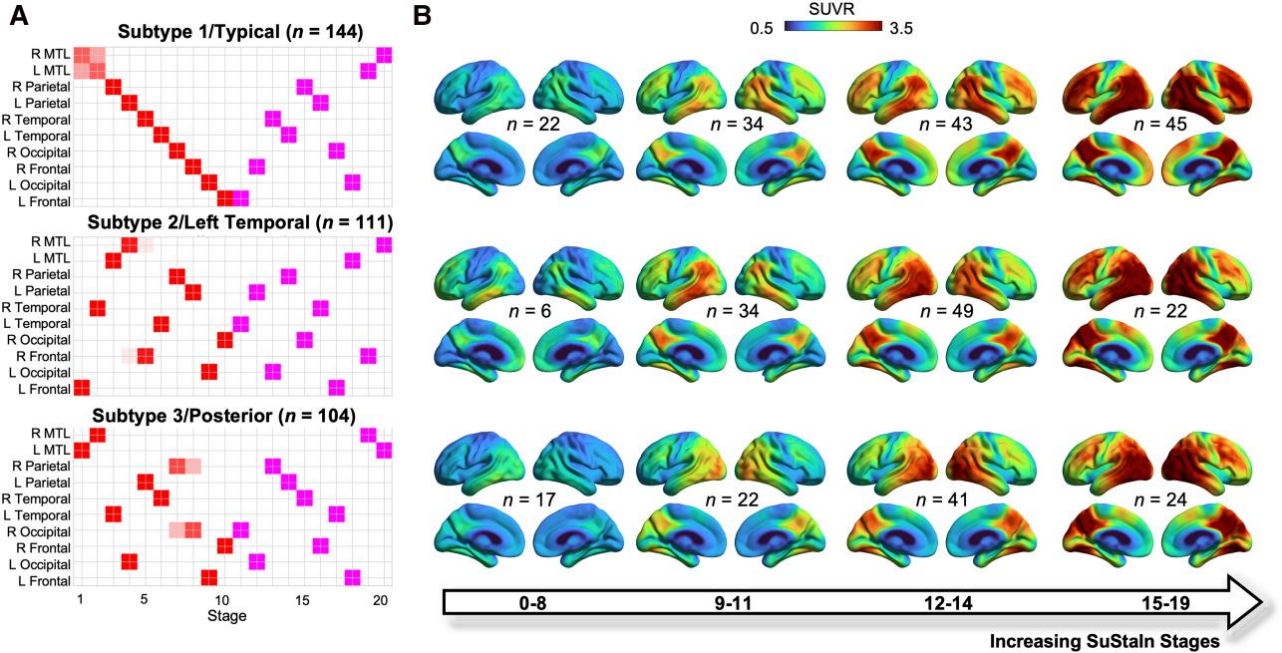
**SuStaIn output.** (**A**) Positional variance plots showing the subtype-specific event sequences derived from SuStaIn modelling. Each column represents a regional event, ordered from earliest (left) to latest (right), with filled blocks indicating the estimated event occurrence and blurriness indicating uncertainty in the event ordering estimation. L: Left; R: right; MTL: medial-temporal lobar ROI including amygdala, hippocampus, and entorhinal cortex. (**B**) Group-average [^18^F]Flortaucipir PET SUVR maps are shown for each subtype across sequential stage bins, as indicated by the bottom arrow (*n* = number of participants per bin).

The event sequences inferred by SuStaIn for each of the three subtypes are presented in Fig. 1A, showing the modelled tau trajectories across ROIs based on the prespecified thresholds. Average [^18^F]Flortaucipir SUVR maps for each subtype and stage group are shown in Fig. 1B. The subtypes are labelled according to their dominant regional involvement: Subtype 1 (S1/Typical, *n* = 144/359, 40%, 95% CI: 35–45%) showed early-stage tau deposition in temporoparietal areas followed by the occipital and frontal areas. Subtype 2 (S2/Left Temporal, *n* = 111/359, 31%, 95% CI: 26–36%) was characterized by asymmetric early-stage tau deposition, predominantly affecting the left temporal lobe. Subtype 3 (S3/Posterior, *n* = 104/359, 29%, 95% CI: 24–34%) displayed a relatively early posterior pattern, with tau burden in the occipital cortex and adjacent temporoparietal areas, which then extended anteriorly towards frontal regions as stage advanced.

**Figure 2:**
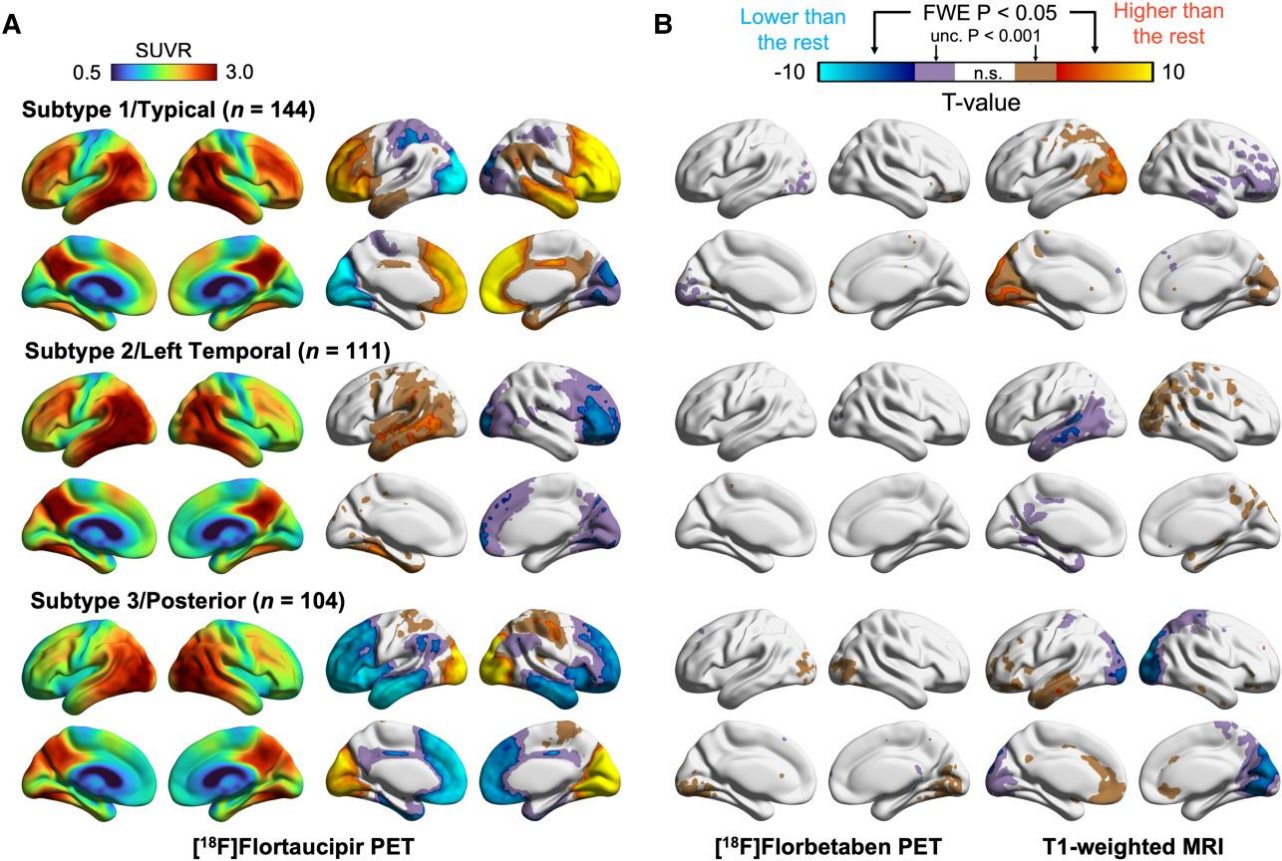
**SuStaIn subtype baseline imaging characteristics.** (**A**) Left column: average [^18^F]Flortaucipir PET SUVR maps. Right column: subtype comparisons for [^18^F]Flortaucipir PET. (**B**) Subtype comparisons for [^18^F]Florbetaben PET (left column), and T1-weighted MRI (right column) at baseline. Voxel-wise statistical comparisons were performed for each subtype versus the rest of the subtypes using general linear models adjusted for baseline age, sex, years of education, SuStaIn stage, and Centiloid (excluded for [^18^F]Florbetaben PET models); MRI models also adjusted for Total Intracranial Volume. Coloured areas in the second to fourth columns indicate regions of statistically significant differences with a double threshold: uncorrected *P* < 0.001 (inside pair) and FWE corrected *P* < 0.05 (outside pair). n.s.: not significant.

Average [^18^F]Flortaucipir SUVR maps and voxel-wise comparisons between each subtype and the rest of the EOAD group further highlighted the distinct tau patterns across subtypes, as well as the corresponding differences in [^18^F]Florbetaben PET and MRI measures (Fig. 2). Compared with the other subtypes, S1/Typical showed significantly higher [^18^F]Flortaucipir binding in bilateral frontal and lateral temporal regions, but significantly lower binding in the occipital regions. Meanwhile, S2/Left temporal showed significantly elevated tau in the left temporal and parietal regions, with lower tau in right frontal and posterior regions. Finally, S3/Posterior exhibited significantly higher tau in occipital and superior parietal regions, with relatively lower binding in frontal and temporal areas bilaterally (FWE *P* < 0.05).

Subtype-versus-rest comparisons for MRI revealed spatial patterns of atrophy that closely mirrored those of [^18^F]Flortaucipir PET, where regions with significantly higher baseline tau signal tended to show significantly greater atrophy (FWE *P* < 0.05), albeit in less extensive areas. Subtle differences were observed in [^18^F]Florbetaben PET comparisons at the uncorrected threshold (*P* < 0.001) but did not persist when corrected for multiple comparisons (FWE *P* < 0.05). Specifically, S3/Posterior demonstrated significantly greater amyloid burden compared with the rest in the occipital region, while S1/Typical was significantly lower compared with the rest in the same region. Baseline subtype-versus-rest comparison with different covariate structures for all modalities showed similar patterns (Supplementary Figs 11 and 12).

### Demographic, clinical, and cognitive profiles of tau subtypes

**Figure 3:**
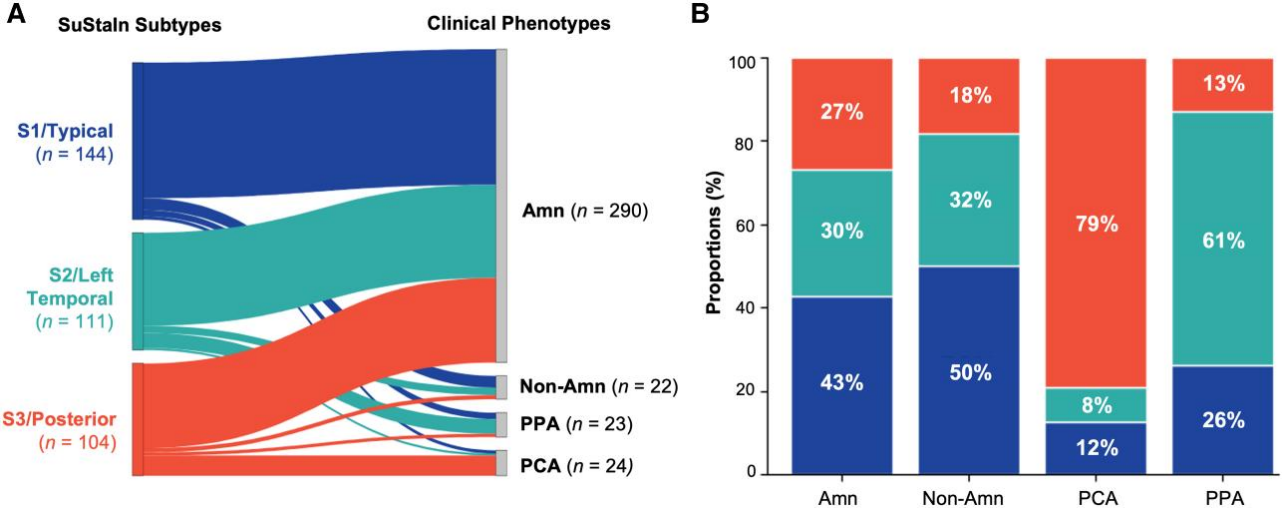
**Subtype baseline relationship with clinical phenotypes.** (**A**) Alluvial plot illustrating the distribution of clinical phenotypes across the three SuStaIn subtypes. For each clinical phenotype shown on the right, *n* denotes the total number of participants within that phenotype. Amn: amnestic predominant cognitive impairment; Non-Amn: non-amnestic predominant cognitive impairment; PPA: primary progressive aphasia; PCA: posterior cortical atrophy. (**B**) Stacked bar charts indicate the proportions of participants within each clinical phenotype being assigned to each of the three SuStaIn subtypes.

As listed in Table 1, no statistically significant differences were found across subtypes in baseline demographics, global amyloid burden (Centiloid), SuStaIn stage, clinical stage, or ApoE4 status (*P* > 0.05). Global tau burden was relatively lower in S3/Posterior. In contrast, SuStaIn subtypes were associated with clinical syndromes. Although most participants with EOAD in LEADS were assigned to a predominantly amnestic phenotype by the site clinician (*n* = 290/359, 81%, 95% CI: 77–85%), this phenotype was significantly overrepresented in S1/Typical (*n* = 124/144, 86%, 95% CI: 81–92%) compared with S3/Posterior (*n* = 78/104, 75%, 95% CI: 67–83%) (*P* < 0.001). As shown in Fig. 3A, there was a significant association between SuStaIn subtypes and clinical phenotypes among those with non-amnestic clinical diagnoses (*n* = 69/359, 19%, 95% CI: 15–23%). Most of the participants (*n* = 14/23, 61%, 95% CI: 41–81%) with PPA were clustered as S2/Left Temporal, and 79% (*n* = 19/24, 95% CI: 63–95%) of participants with PCA were clustered as S3/Posterior (Fig. 3B).

Baseline cognitive performance across subtypes is summarized in Table 1, with post-hoc pairwise comparisons for cognitive tests summarized in Supplementary Table 9. Screening tests of cognition (MMSE and MoCA) and interview-based measures of cognitive function (CDR-SB) were largely similar across subtypes, except for relatively higher MoCA scores in S3/Posterior. When considering additional neuropsychological tests, S3/Posterior showed poorer performance in visuospatial measures, such as the Benson Figure Copy and Line Length tasks. In the Speed/Attention domain, Trail Making Test Part A performance was worse in S3/Posterior and relatively better in S1/Typical, while no significant differences across subtypes were observed for Digit Span Forward. Language measures showed more severe impairments in S2/Left Temporal, particularly on Semantic Fluency, Phonemic Fluency, and Multi-Lingual Naming Test. Other cognitive measures showed limited differences across subtypes.

**Figure 4:**
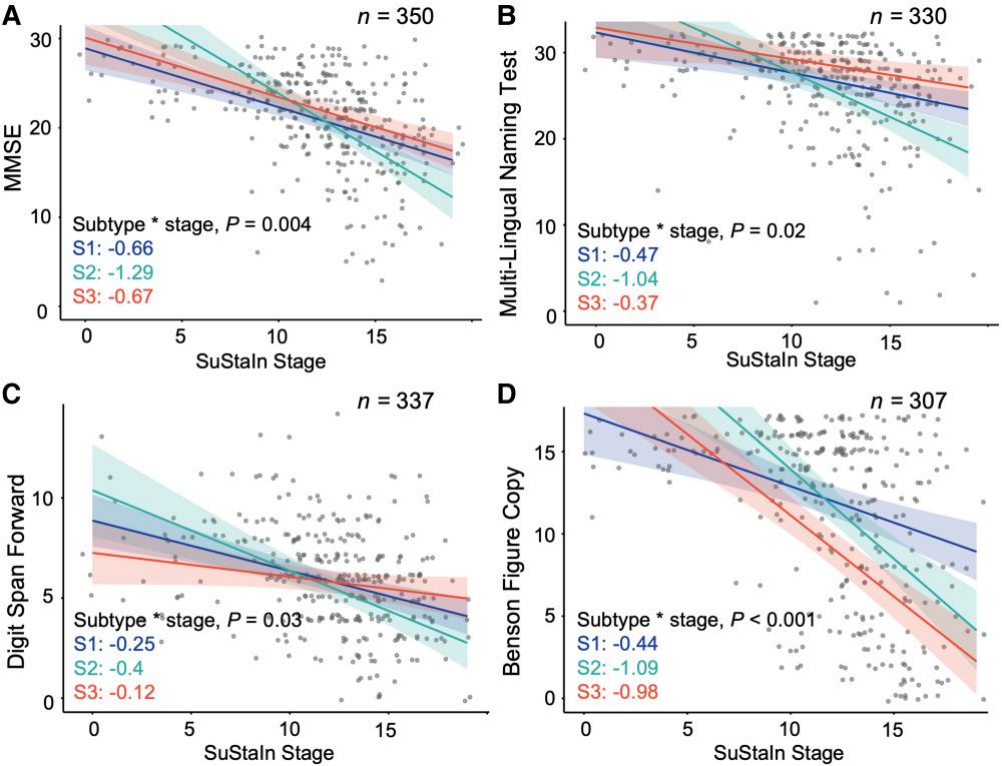
**Baseline stage associations with cognitive scores across subtypes.** Associations between SuStaIn stage and key cognitive measures within each subtype, including (**A**) MMSE, (**B**) Multi-Lingual Naming Test, (**C**) Digit Span Forward, and (**D**) Benson Figure Copy test. Each dot represents one participant’s baseline measurement. The lines represent linear relations estimated with linear regression models, with shaded regions indicating 95% confidence intervals. Each plot also reports the subtype-by-stage interaction *P*-value and the slope for each subtype. All models adjusted for baseline age, sex, years of education, and Centiloid.

At baseline, higher SuStaIn stage was associated with worse cognitive performance across subtypes (Fig. 4). Higher stage correlated with higher CDR-SB scores, although the subtype-by-stage interaction was not significant (*P* = 0.74, S1/Typical: +0.19 CDR-SB/stage; S2/Left Temporal: +0.24 CDR-SB/stage; S3/Posterior: +0.21 CDR-SB/stage). MMSE scores (Fig. 4A) showed a steeper negative association with stage in S2/Left temporal compared with other subtypes. Similarly, the Multi-Lingual Naming Test and Digit Span Forward (Fig. 4B and C) scores showed stronger negative associations with stage in S2/Left Temporal. In contrast, visuospatial impairment measured by the Benson Figure Copy (Fig. 4D) showed weaker association with advancing stage for S1/Typical than the others.

### Longitudinal analysis of disease progression

**Figure 5:**
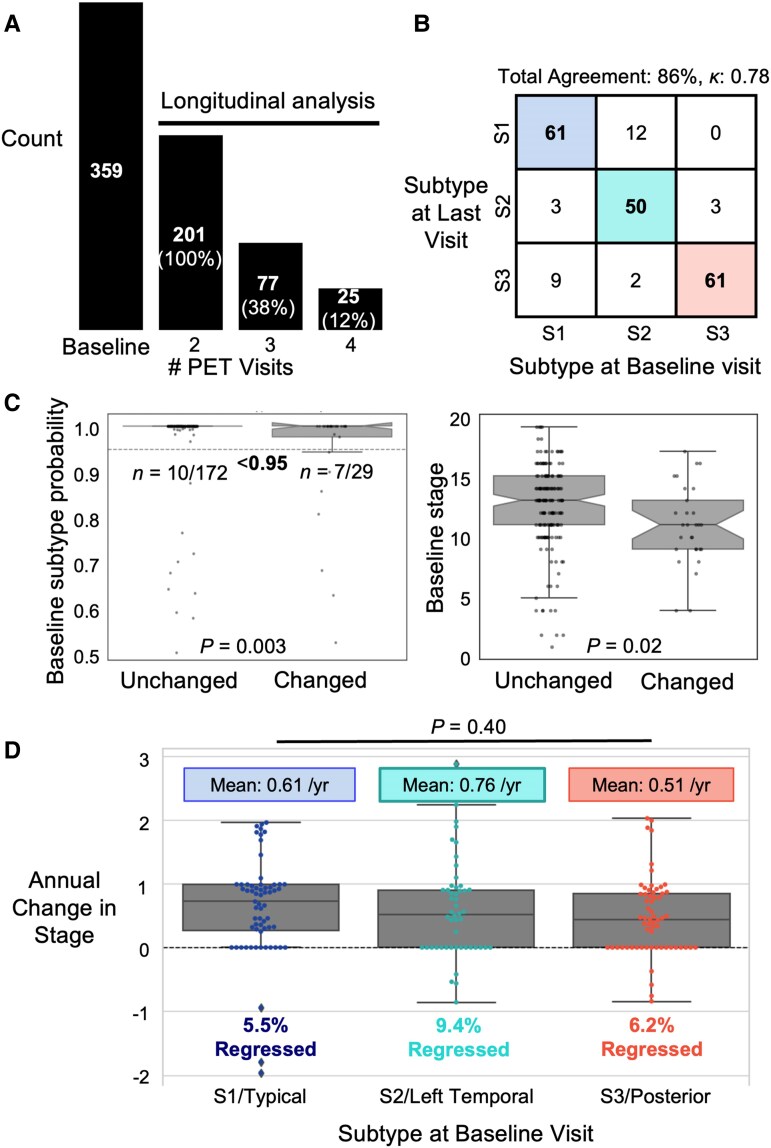
**Subtype stability and stage progression over time.** (**A**) Overview of participants’ follow-up PET visits: *n* = 201 participants had at least one follow-up and contributed to the longitudinal analysis. (**B**) Confusion matrix showing subtype stability between baseline (horizontal axis) and last (vertical axis) [^18^F]Flortaucipir PET visits. Numbers within each cell indicate participant counts. Participants falling within diagonal cells retained their baseline subtype assignments (*n* = 172) and were subsequently analysed for progression within subtypes, as shown in **D**. (**C**) Left: Boxplot of baseline subtype probabilities for participants whose subtype changed versus those who remained stable between first and last visit, compared with chi-squared test using 95% as cut-off. Right: Boxplot of baseline SuStaIn stage for participants whose subtype changed versus those who remained stable, compared with two-sample *t*-test. (**D**) Annual change in SuStaIn stage for each baseline subtype with the mean rates of change indicated above and the percentage of participants who regressed (i.e. showed a decrease in stage between first and last visits) indicated below each box. One-way ANOVA was conducted to compare annual change in stage among subtypes. Each dot represents one participant.

56% (*n* = 201/359, 95% CI: 51–61%) of participants underwent at least one follow-up [^18^F]Flortaucipir PET, with an average follow-up duration of 1.90 ± 0.94 years (Fig. 5A) that did not differ significantly among subtypes (S1/Typical = 1.80 ± 0.93 years; S2/Left Temporal = 1.95 ± 1.03 years; S3/Posterior = 1.97 ± 0.88 years; *F*(2, 198) = 0.65, *P* = 0.52). Participants with follow-up were broadly similar to those with baseline-only data across baseline demographics, neuroimaging measures, cognition, SuStaIn subtype and stage assignment, and disease severity, except for a modestly lower baseline CDR-SB (*P* = 0.052, Supplementary Table 10). Participants with one timepoint were enrolled more recently, with a later median baseline [^18^F]Flortaucipir PET date (baseline-only: May 7, 2022; followed-up: April 5, 2021), consistent with limited follow-up opportunity.

#### Subtype stability and progression over time

Using the SuStaIn model previously trained on baseline data, follow-up [^18^F]Flortaucipir PET scans were subtyped and staged while blinded to participant identity and scan visit. 86% (*n* = 172/201, 95% CI: 81–91%) of participants retained their baseline subtype at the last PET visit (Fig. 5B), with a Cohen’s *κ* of 0.78 (95% CI: 0.70–0.86) between baseline and last-visit subtype assignments, indicating substantial agreement. Compared with participants who remained in the same subtype, those who changed subtype over time—i.e. the off-diagonal cases—had significantly higher proportions of participants classified with low certainty (baseline subtype probability < 95%) (*χ^2^*(1) = 8.5, *P* = 0.003), and lower average baseline SuStaIn stage (*t*(39) = 2.4, *P* = 0.02) (Fig. 5C). Other comparisons between those who changed subtype and those who did not reveal no statistically significant differences and were reported in Supplementary Table 11.

Overall, 94% (*n* = 161/172, 95% CI: 90–97%) of participants, whose longitudinal scans clustered within the same subtype over time as at baseline, either progressed or remained stable in SuStaIn stages. Annual rates of stage change were similar across the three subtypes (*F*(2, 169), *P* = 0.40), with an average rate of 0.56 (95% CI: 0.45–0.66) stage/year (Fig. 5D). Only a small portion (*n* = 11/172, 6%, 95% CI: 3–10%) of participants regressed in SuStaIn stages within each subtype. Subtype stability and progression estimates were comparable when restricting analyses to first versus second visits for participants with ≥ two visits, with slightly higher proportions of participants remaining stable in SuStaIn stage (Supplementary Table 12 and Fig. 13).

#### Baseline tau subtypes are associated with distinct subsequent tau accumulation patterns

**Figure 6:**
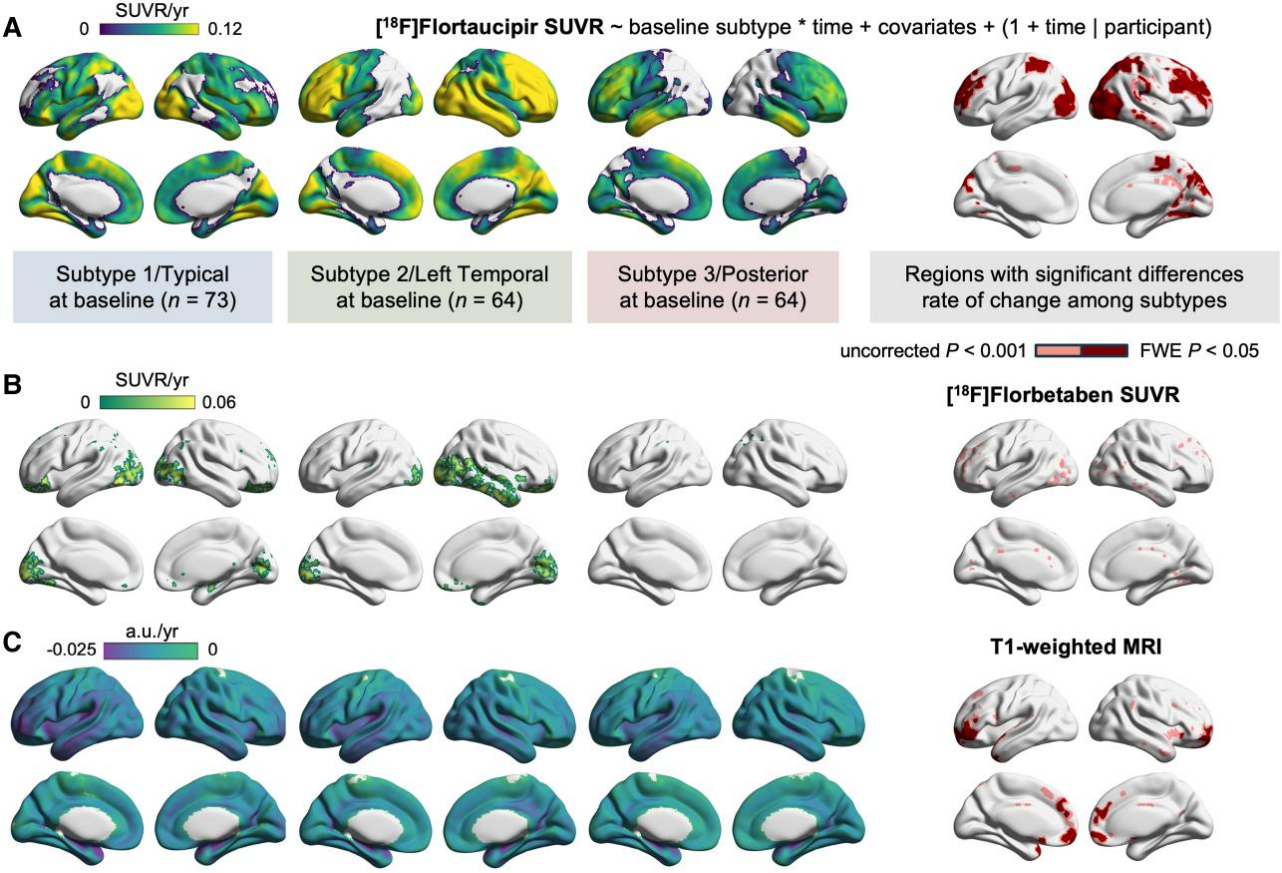
**Longitudinal voxel-wise analysis.** Voxel-wise LME analyses showing subtype-specific annualized rates of change and comparisons among subtypes for (**A**) [^18^F]Flortaucipir PET, (**B**) [^18^F]Florbetaben PET, and (**C**) T1-weighted MRI data. The left three plots in each row display the estimated annualized rate of change, thresholded to show only regions with significant worsening over time within each group (FWE *P* < 0.05). The rightmost plot within each row highlights regions where the rates of change between subtypes were significantly different, using double significance threshold. Voxel-wise LME models included fixed effects for baseline subtype, time from baseline, and their interaction, with covariate adjustment for baseline age, sex, years of education, and Centiloid (excluded for [^18^F]Florbetaben PET models); MRI models also adjusted for TIV. Random intercepts and slopes for time were included for each participant. a.u. = arbitrary unit.

Voxel-wise LME models revealed widespread neocortical increases in [^18^F]Flortaucipir binding over time across all three subtypes, with distinct spatial distributions (Fig. 6A). S1/Typical showed significant extensive increases, sparing only limited areas of the temporoparietal and lateral frontal cortices (FWE *P* < 0.05). S2/Left Temporal exhibited more rapid increases, primarily in the right frontal, temporal, and occipital regions, with no significant change observed in the left lateral temporoparietal regions. S3/Posterior showed increases in frontal, temporal, and medial occipital areas, with no significant change in lateral occipital and parietal regions. Differences in the rates of tau accumulation between subtypes (i.e. interaction term) were significant in regions within the bilateral occipitoparietal, left lateral occipital, and dorsolateral prefrontal regions (FWE *P* < 0.05).

When stratifying by baseline stage within each subtype, both high-stage (>12) and low-stage (≤12) groups showed distinct spatial patterns of tau increase across subtypes. In the low-stage groups, temporo-parietal accumulation was more pronounced in regions that did not survive stringent group-level thresholds when pooled, suggesting that at earlier stages tau continues to accumulate in regions with elevated baseline signal while spreading to additional areas (Supplementary Fig. 14).

#### Baseline tau subtypes are associated with longitudinal amyloid and atrophy patterns

Similar analyses (Fig. 6B) showed increases in amyloid deposition in occipital regions for S1/Typical, bilateral occipital and right temporal regions for S2/Left Temporal, and right inferior parietal region for S3/Posterior (FWE *P* < 0.05). The interaction effect of time by baseline subtype showed differences in limited regions within the occipital and frontal cortices but did not survive correction for multiple comparisons (uncorrected *P* < 0.001, FWE *P* > 0.05).

Similar models for grey matter density showed significant decline in widespread areas for all subtypes. The interaction term showed that differences in patterns of grey matter loss across subtypes were mostly observed in frontal regions, with S1/Typical showing the greatest atrophy rates (Fig. 6C). Supplementary Figs 15 and 16 showed results from LME models without covariate adjustment (aside from TIV for MRI) or additionally adjusting for baseline SuStaIn stage. While the patterns are largely similar, additionally adjusting for SuStaIn seemed to accentuate between-group differences in rates.

#### Differential patterns of global and domain-specific cognitive decline

Differences in rates of global and domain-specific cognitive decline were observed across subtypes (Table 2). For global cognition, both MoCA and CDR-SB declined at different rates, with S3/Posterior exhibiting slower worsening. Significant interactions were also observed for Visuospatial tests (Benson Figure Copy), Speed/Attention (Trail Making Test Part A, Digit Span Forward), Language (Multi-Lingual Naming Test), and Executive function (Trail Making Test Part B, Digit Span Backward). Across these domains, S3/Posterior frequently demonstrated slower decline relative to at least one other subtype, with pairwise comparisons confirming significantly slower decline compared with S2/Left Temporal in Visuospatial, Speed/Attention, and Language tasks, and compared with S1/Typical in Executive functions. In contrast, S2/Left Temporal exhibited steeper decline in Language. Longitudinal trajectories for tests with significant interactions are shown in Supplementary Fig. 17, in complement to Table 2.

**Table 2:** Change in cognition over time across subtypes for cognitive assessments with longitudinal data

Test	*N* (participant)	*n*	S1 Slope	S2 Slope	S3 Slope	Interaction *P*-value	S1 versus S2 *P*-value	S1 versus S3 *P*-value	S2 versus S3 *P*-value
MoCA	171	485	−3.09	−3.45	−2.15	**0**.**001**	0.58	**0**.**02**	**0**.**002**
CDR-SB^+^	198	575	2.32	1.83	1.49	**0**.**005**	0.15	**0**.**005**	0.43
Benson Figure Copy	167	465	−2.81	−2.63	−1.48	**0**.**01**	0.93	**0**.**02**	**0**.**05**
Trail Making Test Part A^+^	135	369	23.3	24.29	14.88	**0**.**04**	0.97	0.12	0.07
Digit Span Forward	175	492	−0.96	−0.99	−0.47	**0**.**004**	0.99	**0**.**02**	**0**.**01**
Multi-Lingual Naming Test	170	466	−2.63	−3.77	−1.42	**<0**.**001**	**0**.**04**	**0**.**02**	**<0**.**001**
Semantic Fluency (Vegetable + Animal)	172	477	−3.88	−4.11	−3.00	0.10			
Trail Making Test Part B^+^	75	201	41.38	35.22	9.78	**0**.**002**	0.86	**0**.**02**	**0**.**05**
Digit Span Backward	170	478	−0.93	−0.70	−0.51	**0**.**009**	0.28	**0**.**01**	0.38
Phonemic Verbal Fluency (L + F)	168	461	−3.57	−2.69	−2.32	0.15			
RAVLT Delayed Recall	145	378	−0.35	−0.70	−0.21	0.14			
Craft Story 21 Delayed Recall	166	464	−1.16	−0.71	−0.75	0.17			
Benson Figure Delayed Recall	198	597	7.99	10.11	8.12	0.77			

Lowercase *n* indicates the total number of longitudinal test observations included in the model; uppercase *N* refers to the number of unique participants contributing data. Slopes represent the annualized rate of change estimated from LME models with fixed effects for baseline subtype, time from baseline, and their interaction, adjusting for baseline sex, age, years of education, and Centiloid. Models also included random intercepts and slopes for each participant. For outcomes showing a significant subtype-by-time interaction, pairwise comparisons were performed to assess differences in the rates of change between subtypes. *P*-values are shown in the three columns to the right with those < 0.05 are in bold. +: Positive slope on this cognitive test indicates worsening performance. Otherwise, negative slope indicates worsening.

### Sensitivity analyses

To compare our findings with those previously reported by Vogel *et al*., we conducted a sensitivity analysis using a four-subtype SuStaIn solution.^9^ After excluding nine participants poorly fitted by the model (i.e. probability of belonging to any subtype < 50%, same as the main analyses), the resulting subtypes largely recapitulated the spatial patterns identified in the three-subtype solution. The additional fourth subtype was characterized by an average [^18^F]Flortaucipir SUVR map with elevated tau in the right temporal regions, contrasting the second subtype in terms of lateralization, but exhibited minimal difference under the corrected threshold when compared with the rest (Supplementary Fig. 18).

The main outcomes from the cross-sectional analyses—including subtype demographic, clinical, cognitive, and imaging characteristics—remained largely unchanged when focusing on the three most prevalent subtypes in the four-subtype solution (Supplementary Table 13).

## Discussion

Sporadic EOAD often presents greater clinical heterogeneity than the late-onset presentation.^6,17,19,22,35^ However, its heterogeneity in tau pattern remains under-characterized, especially in ways that disentangle severity from regional variation. Applying the SuStaIn algorithm on cross-sectional tau PET data, we identified three subtypes with distinct topographies of tau deposition. These data-driven subtypes mapped onto known atypical clinical phenotypes and were associated with differences in cognitive profiles. Over time, most patients remained assigned to the same subtype and progressed to more advanced tau stages, and baseline subtypes forecast different patterns of tau PET changes and cortical atrophy. Together, our findings suggested a tau-PET-based subtyping framework for capturing heterogeneity in EOAD and anticipating subsequent imaging and clinical outcomes, with implications for prognosis and trial design.

Baseline subtype-specific tau PET patterns were associated with clinical phenotypes or syndromes commonly seen in sporadic EOAD. Specifically, the most prevalent subtype S1/Typical showed widespread tau burden with early involvement of the bilateral temporoparietal cortices, aligning with the tau PET pattern reported in most studies of patients with Alzheimer’s disease. S2/Left Temporal exhibited a strongly left-lateralized pattern centred in the temporoparietal region, aligning with tau PET findings in PPA associated with Alzheimer’s disease.^8,36,37^ S3/Posterior showed early tau deposition in occipitoparietal regions, consistent with its enrichment in patients with PCA.^8,38,39^ These patterns emerged despite similar baseline clinical stages, demographics, and global amyloid burden across subtypes, underscoring that differences were primarily driven by topographic heterogeneity rather than disease severity. Importantly, tau-based subtypes were not redundant with these known clinical syndromes: the ‘atypical’ (S2/Left Temporal and S3/Posterior) tau-based subtypes expanded beyond the ‘atypical’ (PPA and PCA) clinical phenotypes and included a majority of participants who did not fulfil criteria for these syndromes. This observation might relate to the stringency of the PPA/PCA clinical criteria: participants with predominant language/visuospatial deficits might not fulfil criteria for PPA/PCA due to co-existing memory deficits at symptom onset, while SuStaIn might classify them as S2/S3. This hypothesis is consistent with a recent study of the LEADS cohort, where a clustering of participants based on patterns of neuropsychological test scores resulted in a classification that correlated with, but was not redundant with, the clinical syndromes assigned based on clinicians’ impression.^40^ This previous study emphasized the heterogeneity of cognitive profiles in LEADS participants described as ‘amnestic’ (81% of amyloid-positive clinically impaired participants), consistent with our findings that these participants were assigned to all three tau-PET-based subtypes, with a mild propensity for S1/Typical. Timing differences between imaging and clinical labels may also play a role: Tau PET captures pathology at imaging time, while labels like PPA and PCA are based on both predominant symptoms at diagnosis and the estimated symptom at disease onset, which are prone to recollection bias. Altogether, these data-driven clustering approaches suggest that the atypical clinical syndromes only represent a limited portion of the broader heterogeneity spectrum in EOAD. Unlike syndrome-based classifications, data-driven tau-PET subtype offers key advantages in its objectivity, independence from knowledge of symptom onset, and ability to reflect current disease burden and neuropsychological impairments to predict future clinical progression.

While we recapitulated the posterior-dominant and lateral-temporal variants reported in the four-SuStaIn-subtype framework by Vogel *et al*.,^9^ notable differences exist between the previous publication and our findings. Specifically, we did not identify one of the original subtypes, the limbic-predominant one, even in sensitivity analyses that included 4–6 subtypes. This discrepancy is likely attributable to our focus on EOAD and inclusion criteria (age at screening < 65) as the limbic-predominant subtype was the oldest of the four identified by Vogel *et al*.^9^ The relationship between older age of onset and limbic-predominant AD is also supported by several independent studies using in vivo imaging or postmortem data to identify patients with predominant medial temporal involvement.^12,41-43^ Interestingly, our four-subtype solution revealed a right-lateralized temporal tau subtype which was identified in Vogel *et al*.’s replication analysis and was attributed to different sample size and composition. More broadly, early-onset patients often exhibit extensive and earlier neocortical involvement alongside widespread network inefficiencies and connectivity disruptions, particularly affecting parietal, temporal, and frontoparietal hub regions critical for cognition.^44-47^ Consistent with these cohort differences, the proportion of participants with poor subtype assignment in our early-onset sample was substantially lower than that reported in prior SuStaIn studies (12–22%),^9,31^ likely due to the high overall tau burden as well as the reduced model complexity inherent to a three-subtype solution.

Over time, most participants retained their baseline subtype and advanced in SuStaIn stage along subtype-consistent trajectories, reinforcing that our subtypes capture meaningful topographic features of tau propagation. Among the small number of participants who changed subtype, transitions followed plausible directions—most commonly from S2/Left Temporal to S1/Typical, reflecting spread beyond the initially asymmetric temporal focus tau. Prior research has highlighted the considerable inter-individual variability in regional progression of tau pathology,^48^ which we observed especially in lateral prefrontal, occipital, and superior parietal regions. Notably, while baseline subtypes predicted distinct longitudinal changes in tau, regions with most elevated baseline tau exhibited minimal further accumulation—e.g. sparing of temporoparietal and prefrontal cortices in S1/Typical, left lateral temporal in S2/Left Temporal, and occipitoparietal areas in S3/Posterior. These saturation effects were also seen in subtype-specific progression patterns across stage groups inferred within baseline. Cortical atrophy followed regional tau burden, with relatively more pronounced baseline degeneration observed in regions exhibiting elevated tau signal. Longitudinally, atrophy became more widespread, but differing rates of decline were observed across regions overlapping with those showing varied rates of tau accumulation, particularly in the anterior temporal and frontal regions. These findings reflected known spatial correlation between tau and atrophy,^49,50^ as well as baseline tau’s association with future regional neurodegeneration.^51,52^ In contrast, although S3/Posterior showed slightly elevated posterior amyloid deposition consistent with its PCA enrichment,^53^ amyloid PET did not account for the observed heterogeneity in tau or clinical presentation, reflecting its typically diffuse neocortical patterns across patients with Alzheimer’s disease.^8^ Instead, tau PET revealed distinct regional patterns that aligned with differences in neurodegeneration and symptom profiles, suggesting tau to be the earliest detectable pathology where subtype differences emerge. Several non-mutually exclusive factors—such as distinct gene variants affecting tau proteostasis^54^ and differences in premorbid or developmental network architecture^55,56^—might underlie the selective regional vulnerability that shapes these tau trajectories.

At baseline, S2/Left Temporal showed marked language deficits, S3/Posterior exhibited relatively more prominent visuospatial impairments, and S1/Typical displayed a broader, non-specific cognitive profile. Longitudinally, participants experienced declines in all cognitive domains, reflecting the natural progression from focal to more generalized impairment.^57^ Consistent with Vogel *et al*., S3/Posterior, with focal occipitoparietal tau at baseline, showed slower cognitive decline, while S2/Left Temporal declined most rapidly and S1/Typical followed closely behind. This aligns with prior findings that the extent of tau pathology across the brain relates to cognitive performance at a similar level as temporal tau across most cognitive domains.^58^ Although regional tau correlates well with neurodegeneration and cognitive decline at the group level, these relationships remain variable across individuals, cortical regions, and cognitive domains, due to additional contributors such as co-pathologies, cognitive reserve, or tau-mediated network disruption.^59-62^ Yet, compared with atrophy- or cognition-based clustering frameworks that typically rely on structural or symptomatic endpoints, our approach defines subtypes directly from tau PET, capturing more upstream pathology with greater spatial specificity. This biologically anchored tau-PET-based subtyping could support early identification of individuals on more aggressive or focal disease trajectories, aiding risk stratification and personalized monitoring. At the moment, trial design increasingly hinges on resolving phenotypic and temporal heterogeneity,^7^ while tau-targeting therapeutics are becoming a central focus of disease-modifying efforts, with tau PET being used as a key outcome.^11^ Incorporating such subtyping into clinical trials could improve sensitivity to treatment effects by minimizing within-arm variability and enabling subtype-specific hypotheses to be tested, particularly when paired with outcome measures tailored to each subtype’s cognitive or neuroimaging profile. Moreover, this framework could provide a useful index of evaluating target engagement by indicating whether the progression is altered relative to the expected tau PET spread.

A major strength of this project is our focus on the LEADS cohort, which represents one of the largest and most comprehensively characterized existing sporadic EOAD samples to date. Its prospective, longitudinal, and multimodal design—including harmonized imaging and cognitive assessments—enabled robust investigations of heterogeneity in an early-onset cohort enriched for atypical presentations. While SuStaIn infers ordinal sequences of biomarker events along a pseudotemporal axis from cross-sectional data, longitudinal follow-up enabled indirect validation: subtype assignments were largely stable over time, and voxel-wise analyses revealed distinct regional tau accumulation patterns. Nonetheless, result interpretations could be influenced by methodological choices, including the use of conventional lobar-level ROIs, z-scoring procedures, and threshold definitions. SuStaIn also assumes monotonic biomarker progression and independent variance across regions, which might oversimplify biological dependencies. To evaluate the robustness of our findings, we conducted sensitivity analyses across multiple z-scoring and thresholding approaches, as well as exclusion criteria for poor model fit. Baseline subtype tau PET patterns and associated characteristics remained stable across these configurations. Looking ahead, simulation-based studies using SuStaIn could help determine whether smaller or more variable subgroups were missed. Additionally, application of progression models that relax these constraints—such as those incorporating flexible event ordering or temporal dependencies—could potentially offer more accurate reconstructions of individual-level trajectories.^11,10^

Beyond model-related considerations, tau PET quantifications could be affected by the tracer’s off-target binding, varying dynamic ranges, and sensitivity to partial volume effects, especially in regions with early or low tau deposition. Non-random missingness in longitudinal cognitive data—often driven by dropout among more impaired participants—can bias estimates of subtype-specific decline. Further limitations include LEADS’ exclusion of patients with corticobasal syndrome, which precludes those with the motor variant of AD.^63^ Our sample also lacks labelling of behavioural or dysexecutive AD, which would fall under the existing tau-based subtypes and potentially emerge as the prevalent clinical phenotype for a separate subtype in alternative solutions.^64-67^ Finally, the dataset was collected across U.S. academic centres with affiliated memory clinics and was predominantly composed of non-Hispanic White participants, potentially limiting generalizability to more diverse clinical populations. Several avenues warrant further work to validate the identified tau PET subtypes in this project. Replication in independent early-onset or mixed-age cohorts would help determine whether the subtypes reflect reproducible patterns. With a growing collection of multimodal data, future work could enhance subtype characterization by incorporating modalities such as functional imaging,^47,55^ fluid biomarkers, and multiomics,^6,54^ to help explain the observed heterogeneity in tau patterns and give insights into pathways leading to similar PET phenotypes. Contextual factors, including early life development,^67^ comorbidities, or medication history from sources like electronic health records or clinical trial datasets,^68,69^ might also offer insights into individual differences in disease susceptibility and presentation. Downstream, as baseline subtype and stage assignments correlate with longitudinal tau accumulation, atrophy, and cognitive decline, future analyses could assess their utility for predicting treatment response in trial settings.

In summary, our findings demonstrate that the data-driven tau-PET-based subtyping provides a powerful framework for delineating biologically meaningful heterogeneity in sporadic EOAD. The identified patterns account for variability in clinical and neuropsychological profiles in EOAD, and tau PET’s ability to forecast atrophy, inform progression, and correlate with clinical outcomes further supports its utility. As biomarker-driven models become central to clinical research and therapeutic development, integrating these subtyping frameworks could enhance prognostic precision and aid in early risk stratification, with important applications for clinical care and trial design.

## Supplementary Material

fcag176_Supplementary_Data

## Data Availability

LEADS data can be requested via https://leads-study.medicine.iu.edu/researchers/leads-data-request-application/. Code for this work is publicly available at https://github.com/rablabservice/EOAD_tauPET_subtype. Full three-dimensional voxelwise NIfTI images of the brain visualizations shown in main figures are publicly available on NeuroVault https://identifiers.org/neurovault.collection:23001.
