# Supplementary material for: Tau topography subtypes account for clinical heterogeneity and longitudinal trajectories in early-onset Alzheimer's disease
Source: Brain Commun. 2026 May 18;8(3):fcag176. doi: 10.1093/braincomms/fcag176 (PMC13234610; doi:10.1093/braincomms/fcag176)
Supplement: fcag176_Supplementary_Data [file fcag176_supplementary_data.docx]

Table of Contents

[Supplementary Method 3](#_Toc221627705)

[1. List of cognitive assessment data 3](#_Toc221627706)

[2. SuStaIn Input and Implementation 3](#_Toc221627707)

[3. Longitudinal modeling 3](#_Toc221627708)

[4. References 4](#_Toc221627709)

[Supplementary Figure 1. 5](#_Toc221627710)

[Supplementary Table 1. 6](#_Toc221627711)

[Supplementary Figure 2. 7](#_Toc221627712)

[Supplementary Figure 3. 8](#_Toc221627713)

[Supplementary Figure 4. 9](#_Toc221627714)

[Supplementary Table 2. 11](#_Toc221627715)

[Supplementary Table 3. 13](#_Toc221627716)

[Supplementary Table 4. 14](#_Toc221627717)

[Supplementary Figure 5. 15](#_Toc221627718)

[Supplementary Figure 6. 16](#_Toc221627719)

[Supplementary Figure 7. 17](#_Toc221627720)

[Supplementary Table 5. 18](#_Toc221627721)

[Supplementary Figure 8. 19](#_Toc221627722)

[Supplementary Table 6. 20](#_Toc221627723)

[Supplementary Table 7. 21](#_Toc221627724)

[Supplementary Figure 9. 22](#_Toc221627725)

[Supplementary Table 8. 23](#_Toc221627726)

[Supplementary Figure 10. 24](#_Toc221627727)

[Supplementary Figure 11. 25](#_Toc221627728)

[Supplementary Figure 12. 26](#_Toc221627729)

[Supplementary Table 9. 27](#_Toc221627730)

[Supplementary Table 10. 28](#_Toc221627731)

[Supplementary Table 11. 29](#_Toc221627732)

[Supplementary Table 12. 30](#_Toc221627733)

[Supplementary Figure 13. 31](#_Toc221627734)

[Supplementary Figure 14. 32](#_Toc221627735)

[Supplementary Figure 15. 33](#_Toc221627736)

[Supplementary Figure 16. 34](#_Toc221627737)

[Supplementary Figure 17. 35](#_Toc221627738)

[Supplementary Figure 18. 36](#_Toc221627739)

[Supplementary Table 13. 37](#_Toc221627740)

# Supplementary Method

## List of cognitive assessment data

The cognitive data for this project includes assessments from the National Alzheimer’s Coordinating Center (NACC) Uniform Data Set 3.0 battery.^1^ Global cognition was assessed using the MMSE (at baseline only), Montreal Cognitive Assessment (MOCA), and CDR, while domain-specific function—encompassing memory, language, executive function, visuospatial abilities, and behavior—was evaluated through both the NACC batteries and additional tests, including the Rey Auditory Verbal Learning Test (RAVLT)^2^ and tasks from the Tablet-based Cognitive Assessment Tools (TabCat).^3^

## SuStaIn Input and Implementation

To both standardize [¹⁸F]Flortaucipir SUVR binding across ROIs for cross-region comparability and define severity thresholds, we applied a two-component Gaussian mixture model (2-GMM) twice to the same distribution of [¹⁸F]Flortaucipir SUVR values for each ROI, using data pooled from both CN individuals and participants with EOAD, as detailed below. The first application was used for standardization, and the second for threshold derivation.

In the first step, a 2-GMM was fitted to the raw SUVR values for each ROI separately. We identified the lower-mean component—assumed to reflect background signal and noise—and used bootstrap resampling (*n* = 5,000) to estimate its mean and standard deviation. These values were then used to transform all SUVR values into z-scores. In the second step, the 2-GMM was re-applied to the z-scored values for the same distribution. Two severity thresholds were derived for each ROI: (1) the intersection point between the two Gaussian components, and (2) the mean of the higher-mean component.

This two-tier thresholding approach yielded two discrete events per ROI, resulting in 20 total events across the ten ROIs, as documented in Supplementary Fig. 2. All GMM were implemented using the GaussianMixture class from scikit-learn v1.2.1.^4^

As sensitivity analyses, we evaluated alternative methods for standardization (inclusion of participants with early-onset non-Alzheimer’s disease in the 2-GMM model, or using the CN group as a reference without GMM), GMM configurations (number of components, initialization, number of iterations), and thresholding strategies (varying the number of thresholds per ROI, or applying fixed z-score cutoffs across all regions),^5–7^ detailed in the Supplementary Fig. 3-4 and Supplementary Table 2-3.

For this project, we implemented the algorithm in Python 3.9.6 using the open-source pySuStaIn package (<https://github.com/ucl-pond/pySuStaIn>).^8^

## Longitudinal modeling

To model longitudinal changes in the level of tau, amyloid, and atrophy according to their baseline tau-PET-based subtype, voxel-wise linear mixed-effects (LME) models were fitted using VoxelStats (MATLAB).^9^ The dependent variables were [¹⁸F]Flortaucipir PET, [^18^F]Florbetaben PET, or MRI data from participants with at least one follow-up visit. For time consistency, MRI and [^18^F]Florbetaben PET scans were mapped to the closest [¹⁸F]Flortaucipir PET visit date. The primary independent variables were years from baseline, dummy-coded baseline SuStaIn subtype, and their interaction. Covariates included baseline age, sex, education, and Centiloid (excluded for [^18^F]Florbetaben PET models); TIV was additionally included in MRI models. Participant-specific random intercepts and slopes for time were included in the models to account for inter- and intra-individual variability in baseline signal and rate of change.

Longitudinal changes in cognitive scores were analyzed using LME models implemented in R with the lme4 (v1.1-35.3) and lmerTest (v3.1-3). All available longitudinal cognitive assessments were included, regardless of whether participants had matching [¹⁸F]Flortaucipir PET visits. Cognitive test scores served as the dependent variables, with fixed effects for time (day from baseline), baseline subtype, and their interaction as the primary independent variables. Covariates included baseline age, sex, years of education, and Centiloid. Random intercepts and slopes were modeled at participant-level. Baseline subtype-by-time interaction terms were compared to assess differences in the rate of cognitive decline across subtypes.

## References

1. Weintraub S, Besser L, Dodge HH, et al. Version 3 of the Alzheimer Disease Centers’ Neuropsychological Test Battery in the Uniform Data Set (UDS). *Alzheimer Dis Assoc Disord*. 2018;32(1):10-17. doi:10.1097/WAD.0000000000000223

2. Bean J. Rey Auditory Verbal Learning Test, Rey AVLT. In: Kreutzer JS, DeLuca J, Caplan B, eds. *Encyclopedia of Clinical Neuropsychology*. Springer New York; 2011:2174-2175. doi:10.1007/978-0-387-79948-3_1153

3. Moskowitz T, Rabinowitz N, Johnson E, et al. The TabCAT Brain Health Assessment: A Highly Efficient and Sensitive Approach to Detecting Very Mild Cognitive Impairment (P5.197). *Neurology*. 2016;86(16_supplement):P5.197. doi:10.1212/WNL.86.16_supplement.P5.197

4. Pedregosa F, Varoquaux G, Gramfort A, et al. Scikit-learn: Machine Learning in Python. Published online 2012. doi:10.48550/ARXIV.1201.0490

5. Young AL, Marinescu RV, Oxtoby NP, et al. Uncovering the heterogeneity and temporal complexity of neurodegenerative diseases with Subtype and Stage Inference. *Nat Commun*. 2018;9(1):4273. doi:10.1038/s41467-018-05892-0

6. Vogel JW, Young AL, Oxtoby NP, et al. Four distinct trajectories of tau deposition identified in Alzheimer’s disease. *Nat Med*. 2021;27(5):871-881. doi:10.1038/s41591-021-01309-6

7. Aksman LM, Oxtoby NP, Scelsi MA, et al. A data-driven study of Alzheimer’s disease related amyloid and tau pathology progression. *Brain*. 2023;146(12):4935-4948. doi:10.1093/brain/awad232

8. Aksman LM, Wijeratne PA, Oxtoby NP, et al. pySuStaIn: A Python implementation of the Subtype and Stage Inference algorithm. *SoftwareX*. 2021;16:100811. doi:10.1016/j.softx.2021.100811

9. Mathotaarachchi S, Wang S, Shin M, et al. VoxelStats: A MATLAB Package for Multi-Modal Voxel-Wise Brain Image Analysis. *Front Neuroinformatics*. 2016;10. doi:10.3389/fninf.2016.00020

# Supplementary Figure 1.


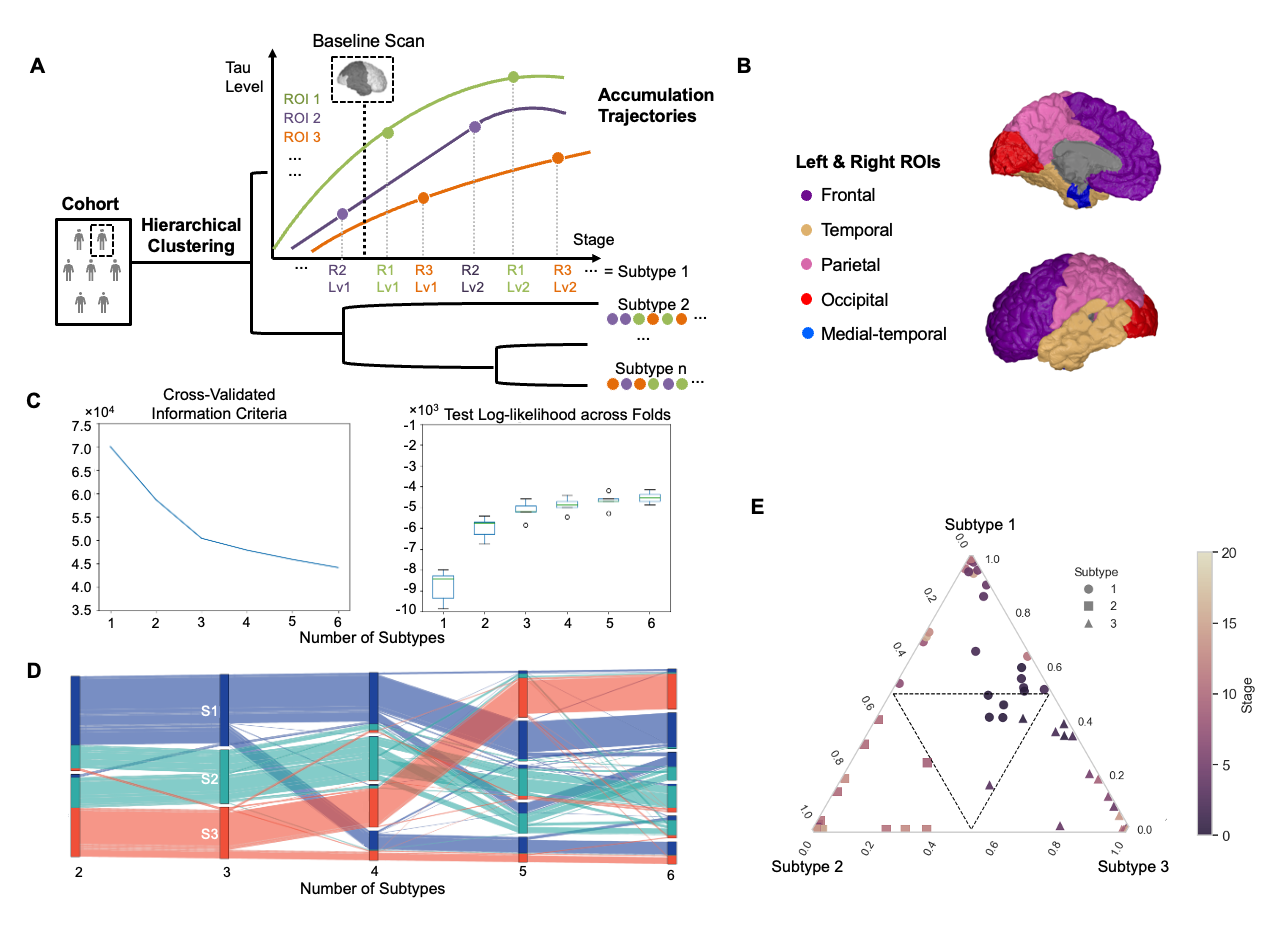


**Supplementary Figure 1. SuStaIn modeling and subtype assignment.** **(A)** Schematic of the SuStaIn model. SuStaIn takes baseline [^18^F]Flortaucipir PET signals across ROIs as input and reconstructs regional tau accumulation trajectories, approximated as threshold-anchored piecewise linear functions. Hierarchical clustering is applied internally to identify subtypes that follow distinct event sequences along the pseudotemporal x-axis, where an “event” represents the tau level crossing a predefined threshold (abbreviated as ‘Lv’ on the x-axis) in a specific region (abbreviated as ‘R’). Participants are then subtyped and staged based on how likely their baseline [^18^F]Flortaucipir PET profiles fit into the subtype-specific sequences. The schematic illustrates example trajectories for three hypothetical ROIs (green, purple, and orange curves) and the resulting subtypes, which differ in the ordering of region-specific tau accumulation events. **(B)** The 10 ROIs defined for this project (five left and five right hemisphere) include the frontal, temporal, parietal, occipital, and medial-temporal lobes. **(C)** Model selection criteria plots: cross-validated information criteria (left; lower values indicate better model fit) and test log-likelihood across folds (right: higher values indicate better generalizability). Together, these measures were used to determine the optimal number of subtypes, favoring simpler models when added complexity brought only minimal improvement. **(D)** Alluvial plot showing participant reassignments across model solutions from two to six subtypes, colored by assignments at the three-subtype solution. **(E)** Ternary scatter plot for the three-subtype solution, showing each participant’s probabilities of belonging to each of the three subtypes. Points within the inner inverse triangle represent participants with low subtype assignment probabilities (<50% for all subtypes), who were considered to have poor model fit and excluded from further analyses. Points are colored by SuStaIn stage, and marker shape indicates final subtype assignment.

# Supplementary Table 1.

| **ROI** | **Parcellations** |
| --- | --- |
| MTL | entorhinal |
|  | amygdala |
|  | hippocampus |
| temporal | superior temporal |
|  | middle temporal |
|  | inferior temporal |
|  | transverse temporal |
|  | parahippocampal |
|  | fusiform |
| frontal | superior frontal |
|  | rostral middle frontal |
|  | caudal middle frontal |
|  | pars opercularis |
|  | pars orbitalis |
|  | pars triangularis |
|  | lateral orbitofrontal |
|  | medial orbitofrontal |
|  | rostral anterior cingulate |
|  | caudal anterior cingulate |
|  | precentral |
|  | insula |
| occipital | lateral occipital |
|  | lingual |
|  | cuneus |
|  | pericalcarine |
| parietal | superior parietal |
|  | inferior parietal |
|  | supramarginal |
|  | postcentral |
|  | precuneus |
|  | posterior cingulate |
|  | isthmus cingulate |

**Supplementary Table 1. Lobar ROI definitions.** The Freesurfer cortical parcellations included in the lobar ROI definitions are listed.

# Supplementary Figure 2.


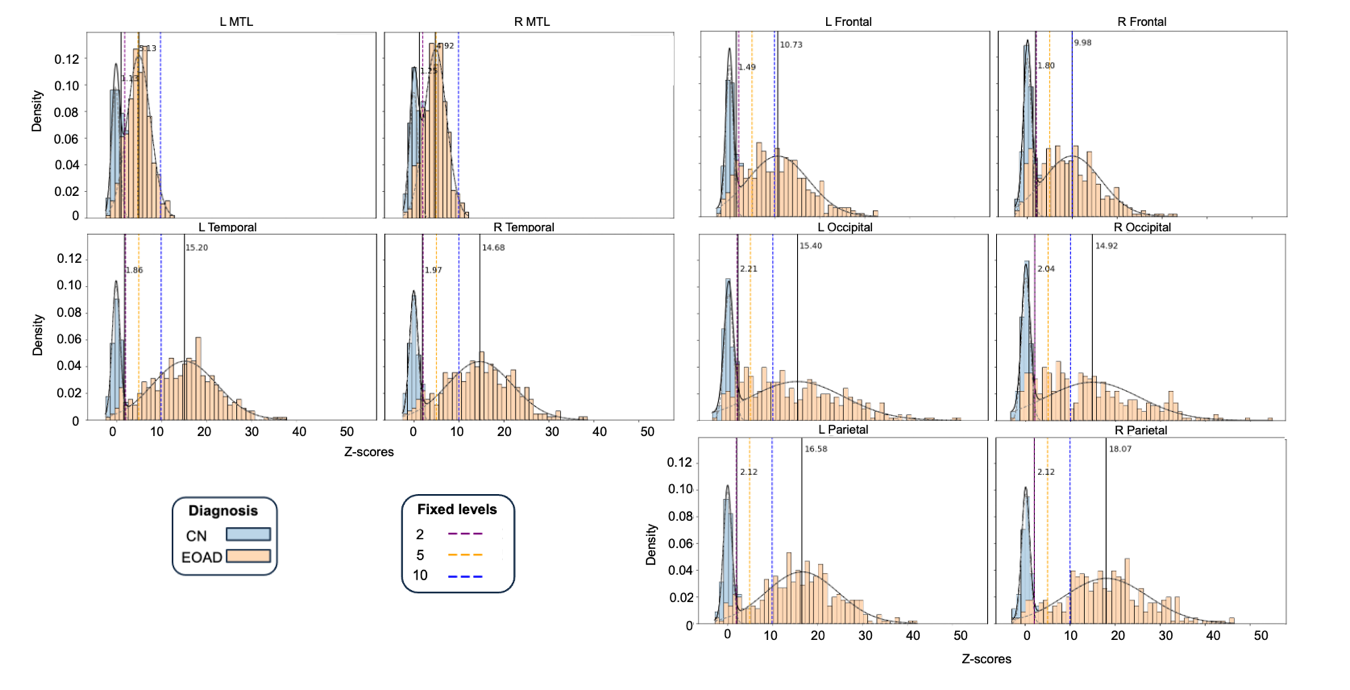


**Supplementary Figure 2. [¹⁸F]Flortaucipir PET z-score distributions and threshold definitions across 10 lobar ROIs**. Histograms show the distribution of z-scores within each of the 10 lobar ROIs across CN (blue shading; *n* = 85) and participants with early-onset Alzheimer’s disease (EOAD; orange shading; *n* = 365). 2-GMM was applied to each distribution to identify a lower-mean and a higher-mean component. The estimated intersection of the two components and the mean of the higher component served as the two thresholds (solid black lines) for each ROI. The fixed levels employed by previous studies are shown using dashed lines (2, purple; 5, yellow; 10, blue).

# Supplementary Figure 3.


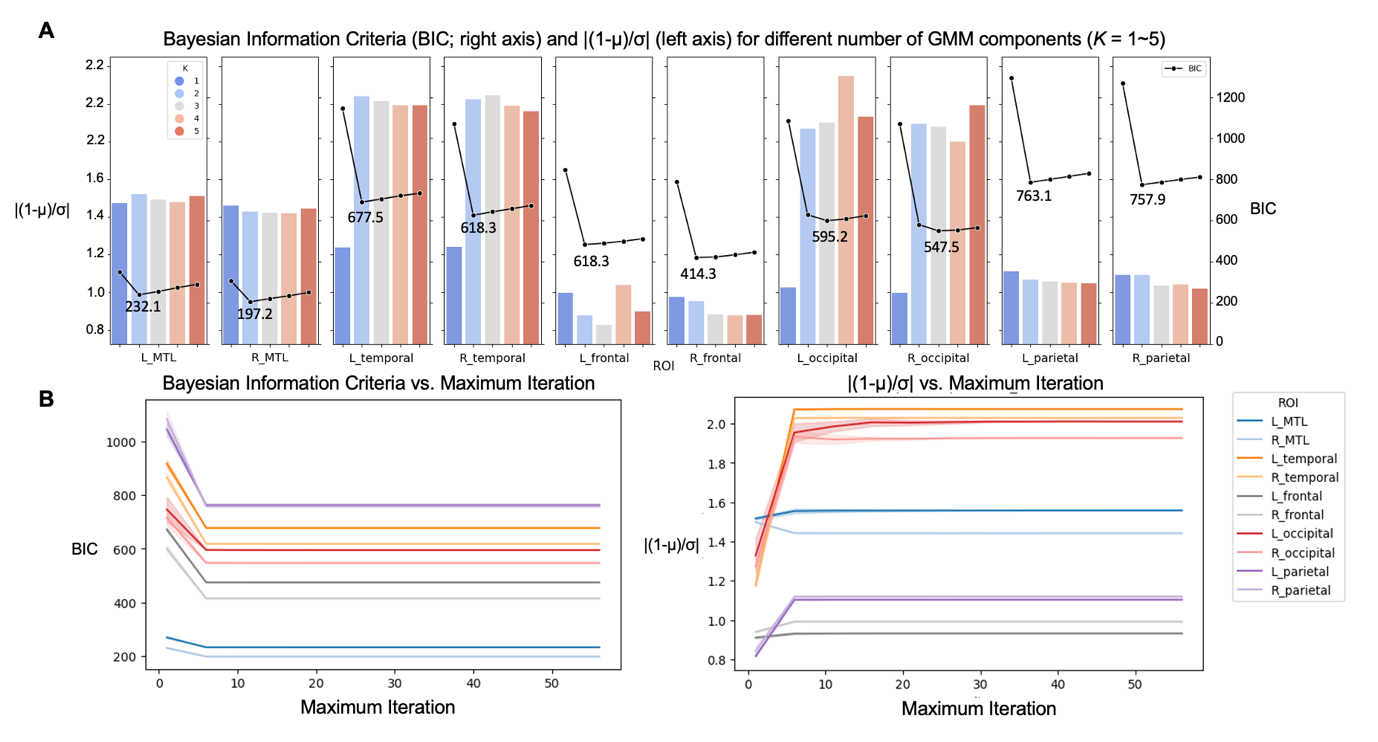


**Supplementary Figure 3. GMM input parameters. (A)** The Bayesian Information Criterion (BIC) and absolute z-ratio ∣(1−μ)/σ∣ (μ, σ being the mean and standard deviation of the lower-mean component from 2-GMM) are plotted across different numbers of GMM components (*K* = 1~5) for each ROI (CN + EOAD participants; *n* = 450). BIC was used to assess overall model fit and complexity, with lower values indicating better fit. Overall, *K* = 2 seemed to be the best for most ROI, except for occipitals where *K* = 3 performed slightly better; but the absolute z-ratios were close between these two, so we opted for *K* = 2 for all ROIs. The optimal BIC is annotated for each ROI. **(B)** Convergence of GMM was evaluated by plotting BIC and absolute z-ratio values across increasing numbers of maximum iterations (max_iter), with tolerance set to 0. Stabilization of both metrics beyond 10 iterations indicated that our choice of setting maximum iteration = 100 would ensure consistent and convergent GMM solutions. Shading indicates standard error across 10 repetitions with different randomizations. All models were run with random initializations instead of the default K-means initializations for consistently lower BIC.

# Supplementary Figure 4.


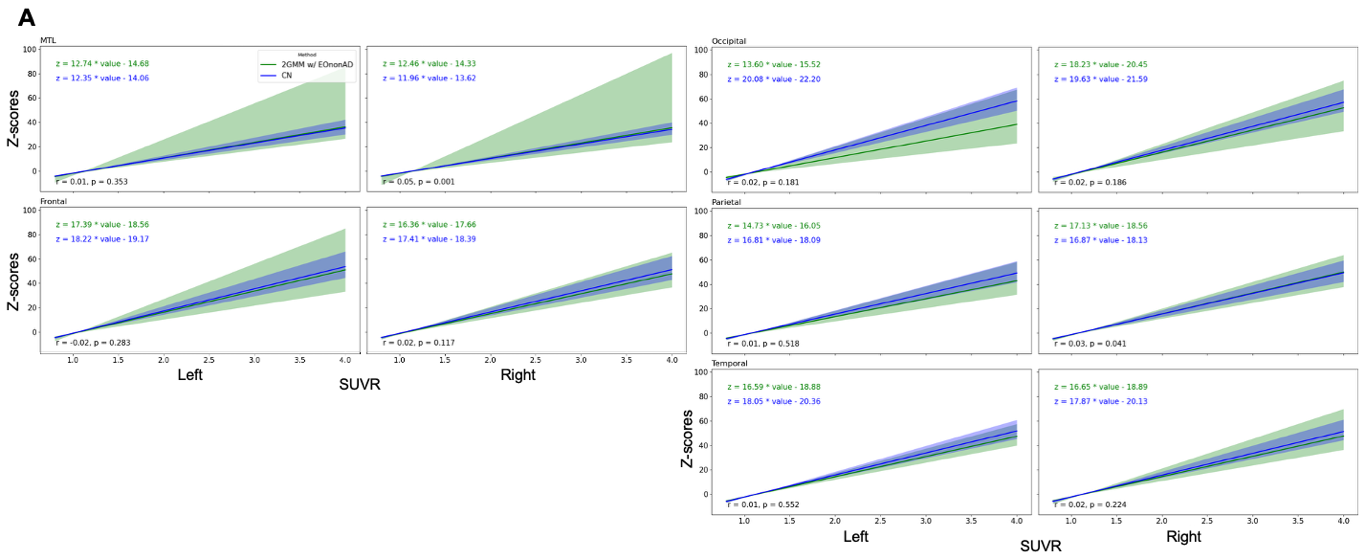

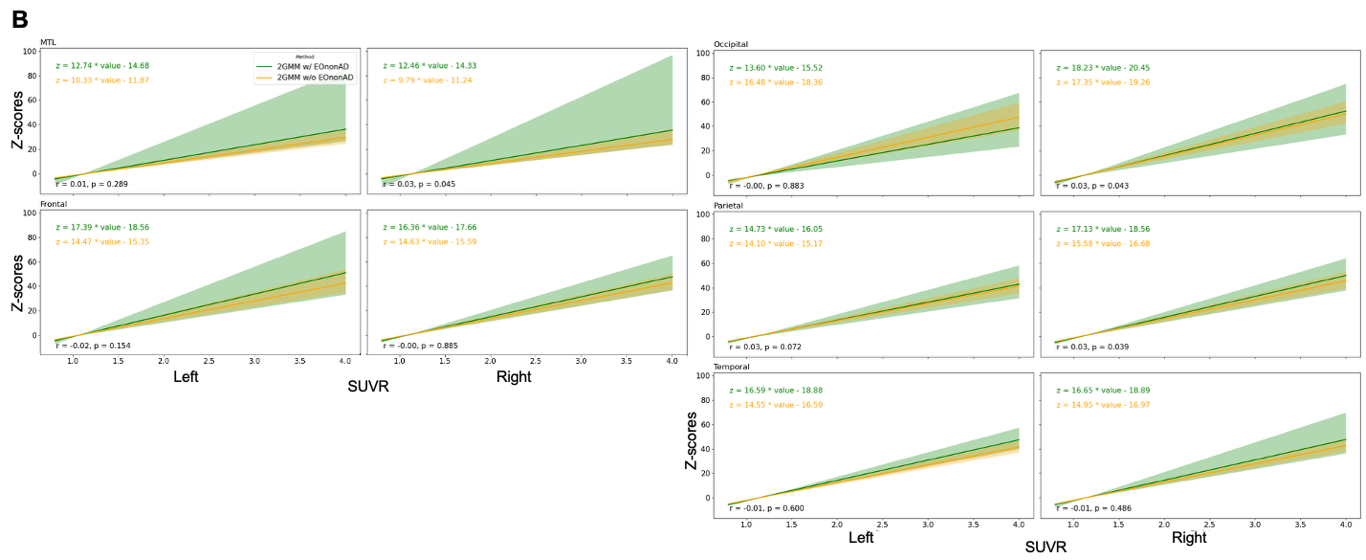

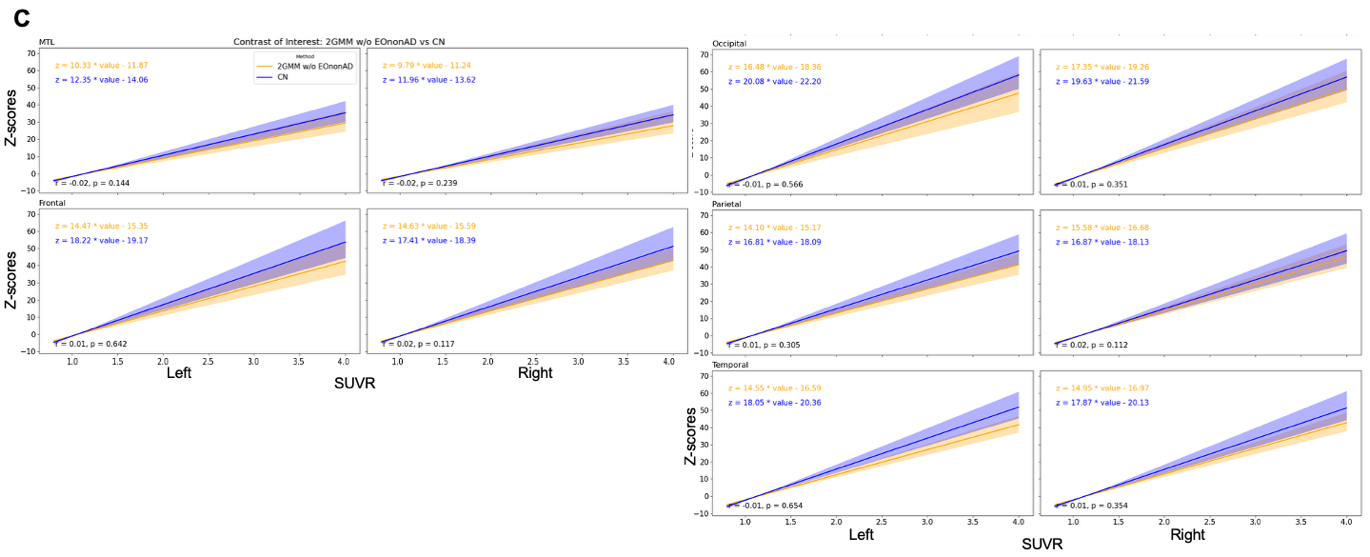


**Supplementary Figure 4. Comparison of z-scoring methods. (A)** Bootstrapped comparisons between z-score transformations derived from 2-GMM fitted on pooled cognitively normal (CN; *n* = 85), early-onset Alzheimer’s disease (EOAD; *n*  = 365), and early-onset non-AD (EOnonAD; *n* = 119) participants (CN+EOAD+EOnonAD, green) versus using only CN participants to standardize (blue). Each subplot corresponds to one ROI and shows the fitted transformation equations for each method as well as the Pearson’s correlation coefficient (*r*) and associated *P*-value between the z-scores derived from the two methods. Shading indicates 95% bootstrap intervals. Observations indicate that including participants with EOnonAD results in generally wider variation in z-scores across bootstrap samples compared to CN standardization alone, likely due to greater heterogeneity in [¹⁸F]Flortaucipir PET signal introduced by non-AD pathology or non-specific binding in EOnonAD cases. **(B)** Bootstrapped comparisons between z-score transformations derived from 2-GMM fitted on pooled CN+EOAD+EOnonAD (green) versus 2-GMM fitted on CN+EOAD (yellow). While the bootstrap-estimated z-score transformations are close between the two methods, observations indicate that excluding participants with EOnonAD from the model fit results in more consistency across samples. **(C)** Bootstrapped comparisons between z-score transformations derived from the CN participants (purple) vs from fitting 2-GMM on pooled CN+EOAD participants (yellow). The CN-method generally results in higher z-score values, but the confidence intervals for both methods overlap substantially. We ultimately opted for the 2-GMM approach between these two because it better captured the underlying distribution of the pooled data.

# Supplementary Table 2.

| **20 stages configuration with alternative definitions for MTL** | | | | | | |
| --- | --- | --- | --- | --- | --- | --- |
|  | **category** | **Intersection/C2 mean** | **MTL only intersection** | **MTL excludes**  **hippocampus** | **MTL excludes hippocampus and intersection only** | |
| number of stages |  | 20 | 18 | 20 | 18 | |
| Subtype stability (% agree, κ) | 1st vs. last | 85.29%, 0.78 | 88.61%, 0.83 | 87.75%, 0.82 | 84.39%, 0.77 | |
|  | 1st vs. 2nd | 87.25%, 0.81 | 93.56%, 0.9 | 89.71%, 0.85 | 86.34%, 0.79 | |
|  | 1st vs. 3rd | 80.00%, 0.69 | 85.9%, 0.78 | 79.75%, 0.69 | 85%, 0.77 | |
| % Regressed | 1st vs. last | 7.35% | 9.41% | 6.86% | 8.29% | |
|  | 1st vs. 2nd | 7.84% | 9.90% | 7.84% | 7.80% | |
|  | 1st vs. 3rd | 6.25% | 6.41% | 5.06% | 8.75% | |
| % Poorly fitted (*n*, %) |  | 6, 1.67% | 7, 1.91% | 8, 2.19% | 1, 0.27% | |
| agreement with PCA (%, *κ)* |  | 82.61%, 0.62 | 73.53%, 0.45 | 82.35%, 0.62 | 78.26%, 0.53 | |
| agreement with PPA (%, *κ*) |  | 73.91%, 0.41 | 73.53%, 0.41 | 70.59%, 0.31 | 76.81%, 0.50 | |
| Stage distribution (*N*, IQR) | 1 | 144, 4 | 133, 4 | 126, 5 | 147, 7 | |
|  | 2 | 111, 3 | 117, 4 | 121, 4 | 110, 3 | |
|  | 3 | 104, 4 | 108, 4 | 110, 4 | 107, 3 | |
| **30 stages configuration with alternative MTL definitions** | | | | | | |
|  | **category** | **Intersection/**  **C2 mean-SD/C2 mean+SD** | **MTL intersection only** | **MTL excludes**  **hippocampus** | **MTL excludes hippocampus and intersection only** | |
| number of stages |  | 30 | 26 | 30 | 26 | |
| Subtype stability (% agree, κ) | 1st vs. last | 85.37%, 0.78 | 80.79%, 0.70 | 80.49%, 0.71 | 79.51%, 0.69 | |
|  | 1st vs. 2nd | 87.32%, 0.81 | 84.73%, 0.77 | 84.39%, 0.76 | 81.95%, 0.73 | |
|  | 1st vs. 3rd | 89.87%, 0.85 | 80.77%, 0.71 | 77.50%, 0.65 | 76.25%, 0.64 | |
| % Regressed | 1st vs. last | 8.78% | 7.88% | 8.78% | 7.80% | |
|  | 1st vs. 2nd | 10.24% | 8.87% | 0.0975 | 7.32% | |
|  | 1st vs. 3rd | 7.59% | 7.69% | 6.25 | 10% | |
| % Poorly fitted (*n*, %) |  | 3, 0.82% | 5, 1.40% | 2, 0.55% | 5, 1.40% | |
| agreement with PCA (%, *κ*) |  | 75.36%, 0.47 | 77.94%, 0.49 | 69.37%, 0.36 | 72.06%, 0.37 | |
| agreement with PPA (%, *κ*) |  | 71.01%, 0.33 | 63.24%, 0.25 | 78.26%, 0.52 | 57.35%, 0.16 | |
| Stage distribution (*N*, IQR) | 1 | 133, 3 | 165, 7 | 137, 7 | 136, 3 | |
|  | 2 | 130, 7 | 111, 6 | 117, 3 | 126, 8 | |
|  | 3 | 99, 6 | 84, 4 | 109, 4 | 98, 3 | |
| **Alternative thresholds** | | | | | |  |
|  | **category** | **Intersection/C2 mean** | **Intersection/C2 mean-SD/C2 mean+SD** | **Fixed (2/5/10)** | **C2 mean-SD/C2mean/C2mean+SD** | |
| number of stages |  | 20 | 30 | 30 | 30 | |
| Subtype stability (% agree, *κ*) | 1st vs. last | 85.29%, 0.78 | 85.37%, 0.78 | 85.85%, 0.79 | 85.5%, 0.78 | |
|  | 1st vs. 2nd | 87.25%, 0.81 | 87.32%, 0.81 | 87.32%, 0.81 | 85.5%, 0.78 | |
|  | 1st vs. 3rd | 80.00%, 0.69 | 89.87%, 0.85 | 83.75%, 0.76 | 78.95%, 0.68 | |
| % Regressed | 1st vs. last | 7.35% | 8.78% | 8.29% | 11.00% | |
|  | 1st vs. 2nd | 7.84% | 10.24% | 7.80% | 12.00% | |
|  | 1st vs. 3rd | 6.25% | 7.59% | 6.25% | 10.53% | |
| % Poorly fitted (*n*, %) |  | 6, 1.67% | 3, 0.82% | 4, 1.10% | 0, 0% | |
| agreement with PCA (%, *κ)* |  | 82.61%, 0.62 | 75.36%, 0.47 | 75.36%, 0.44 | 76.81%, 0.50 | |
| agreement with PPA (%, *κ*) |  | 73.91%, 0.41 | 71.01%, 0.33 | 71.01%, 0.39 | 68.12%, 0.27 | |
| Stage distribution (*N*, IQR) | 1 | 144, 5 | 133, 3 | 138, 3 | 138, 12 | |
|  | 2 | 111, 3 | 130, 7 | 136, 10 | 121, 11 | |
|  | 3 | 104, 4 | 99, 6 | 87, 5 | 106, 7 | |

**Supplementary Table 2.** This table examines how different configurations—number of stages, MTL region definitions, and thresholding— affect the main results. The key criteria assessed include subtype stability (percentage agreement and Cohen’s *κ* between subtype assignments for first vs. last, first vs. second, and first vs. third visits), percentage of participants with stage regression, percentage of participants with poor model fit (subtype assignment probability < 50%), agreement with clinical phenotypes among those with atypical presentations, and stage distribution (median and IQR). Each row of the table represents these metrics for one configuration, with separate panels for 20-stage models, 30-stage models, and alternative threshold definitions. C2 = higher-mean component from fitting 2-GMM onto the z-scores. Intersection = higher- and lower-mean component intersection. “MTL intersection only” indicates only setting one threshold for both the right and left MTL ROI. “MTL excludes hippocampus” indicates defining the MTL regions to include only the hippocampus and amygdala. These alternative configurations were attempted due to the inconsistency observed in these two ROIs for the event sequences (see Supplementary Fig. 6), but they did not address the problem. Across most configurations, high subtype stability (typically >80% agreement) and moderate to strong *κ* values for subtype assignment agreement (0.7–0.85) were observed between baseline and follow-up visits. Regression percentages and percentages of participant with poor model fit were mostly low (mostly <10%), while agreement with PCA and PPA diagnoses varied moderately depending on configurations. Median SuStaIn stage and IQR values showed some variability across configurations but are largely driven by the number of stages. We retained the intersection/C2 mean thresholding and original ROI definitions due to the relatively good agreement between clinical phenotypes and SuStaIn subtypes as well as reasonable longitudinal stability and progression.

# Supplementary Table 3.

| **2-GMM Configuration** | |
| --- | --- |
| Number of components | 2 |
| Initialization method | Random data points |
| Number of random initializations | 100 |
| Maximum EM iterations | 300 |
| Convergence tolerance | 0 |
| **SuStaIn Configuration** | |
| Input data | Baseline [¹⁸F]Flortaucipir PET from 365 participants with EOAD |
| Features | Z-scored SUVR across 10 lobar ROIs with ROI-specific thresholds |
| Maximum number of subtypes explored | 6 |
| Sequence optimization start points | 25 random starts |
| Maximum z-score per ROI | 95th percentile of empirical distribution |
| MCMC sampling iterations | 1,000,000 |
| Poor model fit definition | Subtype probability < 50% (excluded from analyses) |

**Supplementary Table 3. Final SuStaIn and 2-GMM configurations.** See supplementary method section for package and version information. EM: Expectation Maximization algorithm.

# Supplementary Table 4.

| **Variables** | **Cognitively Normal Participants (*n* = 85)** |
| --- | --- |
| Years of Education | 16.7 (2.0) |
| Age | 56.7 (5.9) |
| Sex – Female | 54 (63.5%) |
| ApoE4 Carrier | 33 (38.8%) |
| [¹⁸F]Florbetaben, Centiloid | 2.3 (7.4) |
| [¹⁸F]Flortaucipir SUVR, all cortical ROIs | 1.1 (0.1) |
| [¹⁸F]Flortaucipir SUVR, temporal Meta ROI | 1.1 (0.1) |
| MoCA | 27.2 (2.2) |
| CDR-SB | 0.0 (0.1) |
| MMSE | 29.2 (0.9) |

**Supplementary Table 4. CN participant characteristics.** For numeric variables, mean (SD); for categorical variable, raw count (percentage).

# Supplementary Figure 5.


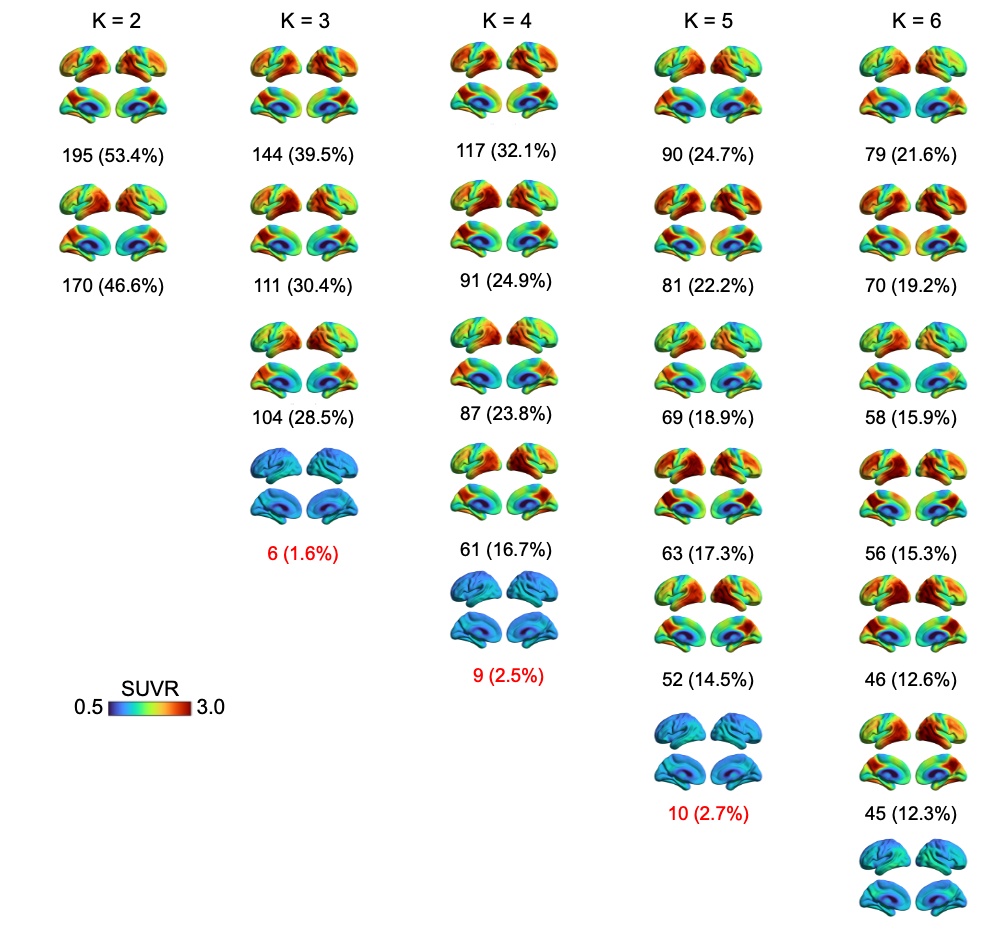


**Supplementary Figure 5. Group-average [^18^F]Flortaucipir PET SUVR images across solutions** with number of subtypes (*K*) = 2 ~ 6 (left to right). Within each solution (column), subtypes are sorted by most to least prevalent (top to bottom), where number and percentage of participants being assigned to each subtype are indicated below. The additional group shown at the bottom of each column with red text represents the average image for participants with poor model fit (probabilities of belonging to any subtype < 50%).

# Supplementary Figure 6.


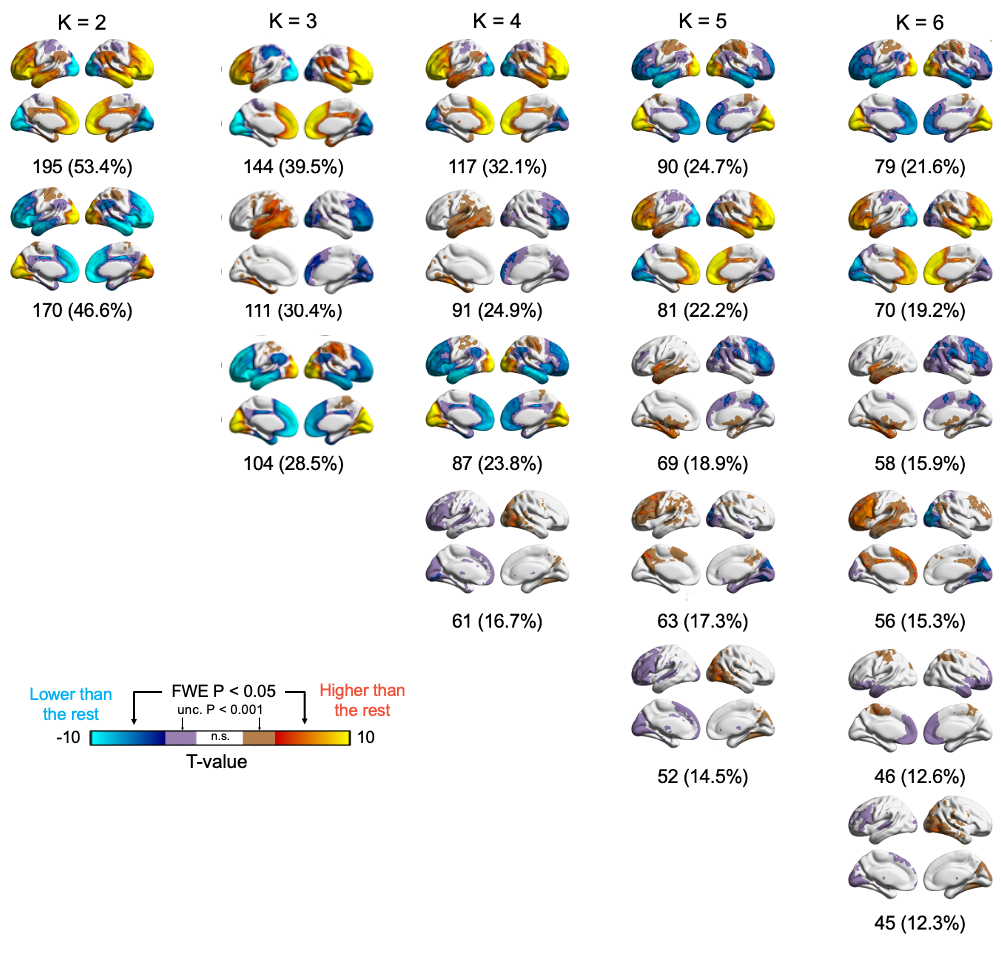


**Supplementary Figure 6.** Subtype-versus-rest [^18^F]Flortaucipir PET voxel-wise comparisons across solutions with number of subtypes (*K*) = 2 ~ 6 (left to right), performed with general linear models adjusted for baseline age, sex, years of education, SuStaIn stage, and Centiloid. Colored areas indicate regions of statistically significant differences with a double threshold: uncorrected *P* < 0.001 (pale colors) and FWE corrected *P* < 0.05 (bright colors). n.s.: not significant. Within each solution (column), subtypes are sorted by most to least prevalent (top to bottom), where number and percentage of participants being assigned to each subtype are indicated below.

# Supplementary Figure 7.

**
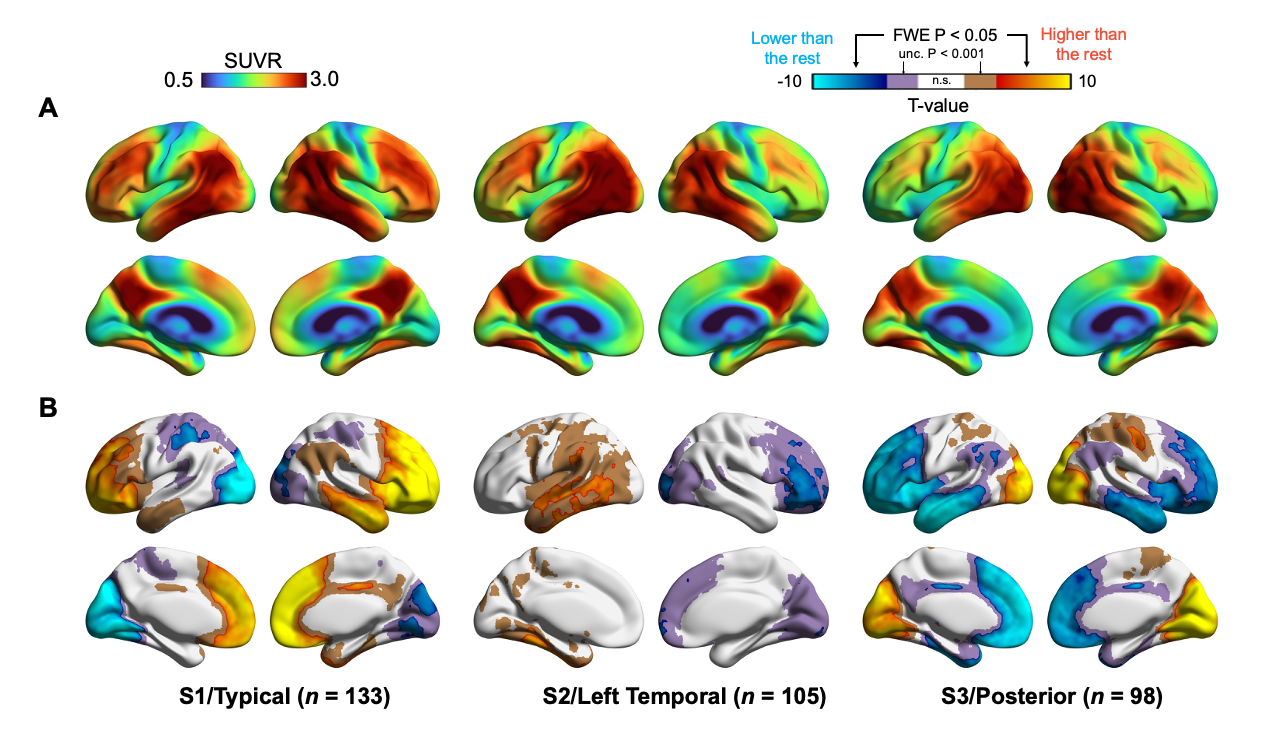
**

**Supplementary Figure 7. Voxel-wise baseline comparisons with 80% probability exclusion for poor model fit.** Voxel-wise baseline comparisons after excluding participants with probability belonging to any subtype below 80%. **(A)** Average [¹⁸F]Flortaucipir PET SUVR images. **(B)** Subtype-versus-rest comparisons using general linear models adjusted for baseline age, sex, years of education, SuStaIn stage, and Centiloid. Colored areas indicate regions of statistically significant differences with a double threshold: uncorrected *P* < 0.001 (pale colors) and FWE corrected *P* < 0.05 (bright colors). n.s.: not significant. Number of participants being assigned to each subtype is indicated below each column.

# Supplementary Table 5.

| **Variables** |  | **Missing** | **Total**  **(*n* = 336)** | **S1/Typical**  **(*n* = 133)** | **S2/Left Temporal (*n* = 105)** | **S3/Posterior**  **(*n* = 98)** | ***P-*Value** |
| --- | --- | --- | --- | --- | --- | --- | --- |
| **Demographics** |  |  |  |  |  |  |  |
| Years of Education |  | 0 | 15.7 (2.4) | 15.6 (2.5) | 15.6 (2.5) | 15.9 (2.4) | 0.62 |
| Age |  | 0 | 59.0 (4.0) | 58.7 (4.1) | 58.8 (3.9) | 59.4 (3.9) | 0.36 |
| Sex – Female |  | 0 | 187 (55.7) | 73 (54.9) | 60 (57.1) | 54 (55.1) | 0.93 |
| ApoE4 Carrier |  | 8 | 183 (54.5) | 67 (50.4) | 57 (54.3) | 59 (60.2) | 0.67 |
| **Neuroimaging** |  |  |  |  |  |  |  |
| [¹⁸F]Florbetaben, Centiloid |  | 0 | 104.4 (27.4) | 105.9 (29.0) | 103.0 (25.0) | 103.8 (27.8) | 0.69 |
| [¹⁸F]Flortaucipir SUVR (all cortical ROIs) |  | 0 | 2.0 (0.4) | 2.0 (0.5) | 2.0 (0.3) | 1.9 (0.4) | **0.05** |
| [¹⁸F]Flortaucipir SUVR (temporal Meta ROI) |  | 0 | 2.3 (0.5) | 2.3 (0.5) | 2.3 (0.4) | 2.1 (0.4) | **<0.001** |
| SuStaIn Stage |  | 0 | 12.6 (3.2) | 12.6 (3.6) | 12.7 (2.6) | 12.4 (3.2) | 0.76 |
| **Clinical** |  |  |  |  |  |  |  |
| Diagnosis – Dementia |  | 1 | 256 (76.2) | 103 (77.4) | 79 (75.2) | 74 (75.5) | 0.63 |
| Clinical Phenotype, *n* (%) | Amnestic | 0 | 269 (80.1) | 114 (85.7) | 83 (79.0) | 72 (73.5) | **<0.001** |
|  | Non-amnestic |  | 20 (6.0) | 10 (7.5) | 6 (5.7) | 4 (4.1) |  |
|  | PCA |  | 24 (7.1) | 3 (2.3) | 2 (1.9) | 19 (19.4) |  |
|  | PPA |  | 23 (6.8) | 6 (4.5) | 14 (13.3) | 3 (3.1) |  |
| MoCA |  | 26 | 15.2 (6.1) | 14.9 (6.4) | 14.4 (6.2) | 16.6 (5.5) | **0.04** |
| CDR-SB |  | 3 | 4.0 (2.0) | 4.3 (2.3) | 3.8 (1.8) | 3.9 (1.7) | 0.12 |
| MMSE |  | 9 | 21.0 (5.3) | 20.8 (5.7) | 20.5 (5.6) | 21.9 (4.5) | 0.15 |

**Supplementary Table 5. Sensitivity analysis with subject exclusion criteria set to subtype assignment probability < 80%.** Summary of demographic, clinical, and cognitive variables for each of the three subtypes and group comparisons. See Table 1 for details.

# Supplementary Figure 8.

**
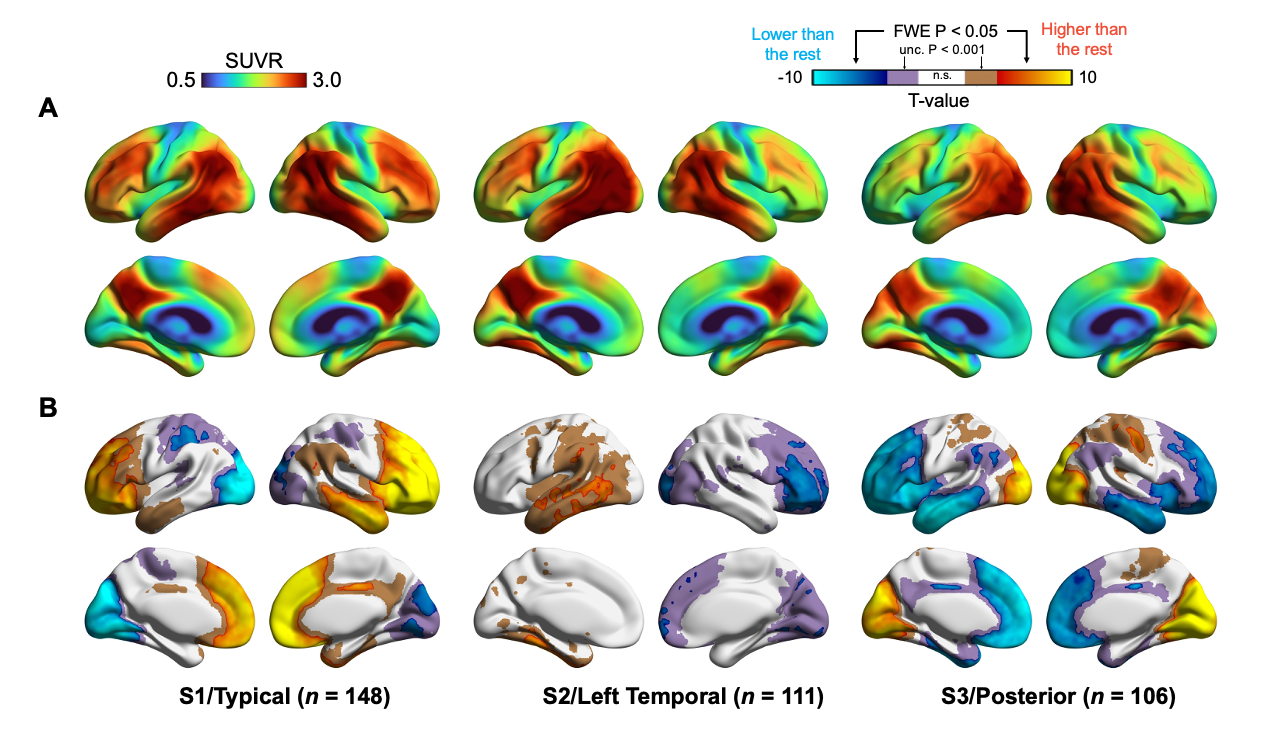
**

**Supplementary Figure 8. Voxel-wise baseline comparisons with no exclusion of participants having poor model fit**. **(A)** Average [¹⁸F]Flortaucipir PET SUVR maps. **(B)** Subtype-versus-rest comparisons using general linear models adjusted for baseline age, sex, years of education, SuStaIn stage, and Centiloid. Colored areas indicate regions of statistically significant differences with a double threshold: uncorrected *P* < 0.001 (pale colors) and FWE corrected *P* < 0.05 (bright colors). n.s.: not significant. Number of participants being assigned to each subtype is indicated below each column.

# Supplementary Table 6.

| **Variables** |  | **Missing** | **Total**  **(*n* = 365)** | **S1/Typical**  **(*n* = 148)** | **S2/Left Temporal (*n* = 111)** | **S3/Posterior**  **(*n* = 106)** | ***P-*Value** |
| --- | --- | --- | --- | --- | --- | --- | --- |
| **Demographics** |  |  |  |  |  |  |  |
| Years of Education |  | 0 | 15.6 (2.4) | 15.6 (2.5) | 15.6 (2.4) | 15.8 (2.4) | 0.72 |
| Age |  | 0 | 59.2 (4.0) | 59.0 (4.1) | 58.9 (3.9) | 59.7 (3.9) | 0.35 |
| Sex – Female |  | 0 | 201 (55.1) | 80 (54.1) | 63 (56.8) | 58 (54.7) | 0.91 |
| ApoE4 Carrier |  | 9 | 200 (54.8) | 74 (50.0) | 59 (53.2) | 67 (63.2) | 0.34 |
| **Neuroimaging** |  |  |  |  |  |  |  |
| [¹⁸F]Florbetaben, Centiloid |  | 0 | 102.5 (28.1) | 103.0 (29.7) | 103.3 (24.5) | 100.8 (29.4) | 0.76 |
| [¹⁸F]Flortaucipir SUVR  (all cortical ROIs) |  | 0 | 1.9 (0.4) | 1.9 (0.5) | 2.0 (0.3) | 1.8 (0.4) | **0.04** |
| [¹⁸F]Flortaucipir SUVR  (temporal Meta ROI) |  | 0 | 2.2 (0.5) | 2.2 (0.6) | 2.3 (0.4) | 2.0 (0.5) | **<0.001** |
| SuStaIn Stage |  | 0 | 12.0 (4.0) | 11.8 (4.6) | 12.6 (2.6) | 11.6 (4.1) | 0.15 |
| **Clinical** |  |  |  |  |  |  |  |
| Diagnosis – Dementia |  | 1 | 271 (74.2) | 111 (75.0) | 84 (75.7) | 76 (71.7) | 0.59 |
| Clinical Phenotype, *n* (%) | Amnestic | 0 | 296 (81.1) | 128 (86.5) | 88 (79.3) | 80 (75.5) | **<0.001** |
|  | Non-amnestic |  | 22 (6.0) | 11 (7.4) | 7 (6.3) | 4 (3.8) |  |
|  | PCA |  | 24 (6.6) | 3 (2.0) | 2 (1.8) | 19 (17.9) |  |
|  | PPA |  | 23 (6.3) | 6 (4.1) | 14 (12.6) | 3 (2.8) |  |
| MoCA |  | 28 | 15.6 (6.1) | 15.4 (6.4) | 14.5 (6.1) | 17.0 (5.6) | **0.02** |
| CDR-SB |  | 3 | 3.9 (2.0) | 4.1 (2.3) | 3.7 (1.8) | 3.7 (1.8) | 0.26 |
| MMSE |  | 9 | 21.3 (5.4) | 21.2 (5.7) | 20.6 (5.5) | 22.3 (4.6) | 0.05 |

**Supplementary Table 6. Sensitivity analysis with subject exclusion criteria set to no exclusion based on model fit.** Summary of demographic, clinical, and cognitive variables for each of the three subtypes and group comparisons. See Table 1 for details.

# Supplementary Table 7.

|  |  | **0.5** | | | **0.8** | | | |
| --- | --- | --- | --- | --- | --- | --- | --- | --- |
|  |  | **non-excluded** | **excluded** | ***P*-Value** | | **non-excluded** | **excluded** | ***P*-Value** |
| *n* |  | 359 | 6 |  | | 336 | 29 |  |
| **Demographics** |  |  |  |  | |  |  |  |
| Years of Education |  | 16.0 [14.0,18.0] | 15.0 [13.2,16.0] | 0.43 | | 16.0 [14.0,18.0] | 16.0 [14.0,16.0] | 0.51 |
| Age |  | 59.3 [56.5,62.4] | 64.5 [64.2,64.8] | **0.002** | | 59.0 [56.3,62.3] | 62.4 [59.9,64.2] | **<0.001** |
| Sex - Female |  | 198 (55.2) | 3 (50.0) | 1 | | 187 (55.7) | 14 (48.3) | 0.57 |
| ApoE4 Carrier |  | 195 (54.3) | 5 (83.3) | 0.36 | | 183 (54.5) | 17 (58.6) | 0.83 |
| **Neuroimaging** |  |  |  |  | |  |  |  |
| [¹⁸F]Florbetaben, Centiloid |  | 103.8 [83.9,121.3] | 64.7 [59.9,70.5] | **0.001** | | 104.0 [85.9,122.3] | 71.4 [60.5,101.9] | **<0.001** |
| [¹⁸F]Flortaucipir SUVR, all cortical ROIs |  | 1.9 [1.6,2.2] | 1.1 [1.0,1.1] | **<0.001** | | 1.9 [1.7,2.2] | 1.2 [1.1,1.6] | **<0.001** |
| [¹⁸F]Flortaucipir SUVR, temporal Meta ROI |  | 2.2 [1.9,2.5] | 1.2 [1.1,1.2] | **<0.001** | | 2.3 [1.9,2.6] | 1.4 [1.2,1.7] | **<0.001** |
| SuStaIn Stage |  | 13.0 [10.0,15.0] | 0.0 [0.0,0.0] | **<0.001** | | 13.0 [11.0,15.0] | 2.0 [0.0,10.0] | **<0.001** |
| **Clinical** |  |  |  |  | |  |  |  |
| MoCA |  | 16.0 [11.0,20.0] | 20.0 [19.2,20.8] | 0.08 | | 16.0 [11.0,20.0] | 21.0 [17.0,22.0] | **<0.001** |
| MMSE |  | 22.0 [18.0,25.0] | 27.5 [26.2,28.0] | **0.005** | | 22.0 [18.0,25.0] | 26.0 [23.0,29.0] | **<0.001** |
| CDR-SB |  | 4.0 [2.5,5.0] | 2.0 [1.5,2.9] | **0.04** | | 4.0 [2.5,5.0] | 2.5 [1.5,3.0] | **<0.001** |
| Diagnosis - Dementia |  | 91 (25.3) | 2 (33.3) | 0.90 | | 79 (23.5) | 14 (48.3) | **0.01** |
| Clinical Phenotype | Amnestic | 290 (80.8) | 6 (100.0) | 0.70 | | 269 (80.1) | 27 (93.1) | 0.20 |
|  | Non-amnestic | 22 (6.1) |  |  | | 20 (6.0) | 2 (6.9) |  |
|  | PCA | 24 (6.7) |  |  | | 24 (7.1) |  |  |
|  | PPA | 23 (6.4) |  |  | | 23 (6.8) |  |  |

**Supplementary Table 7. Comparisons between excluded and non-excluded participants based on model fit.** “0.5” tab indicates that the comparisons are between participants excluded due to having subtype probability < 0.5 vs. non-excluded participants. Likewise for the “0.8” tab. *P*-values are derived from Mann-Whitney U tests and interquartile ranges are reported for continuous variables. Missingness: 9 in MMSE; 28 in MoCA; 1 in Diagnosis; 9 in ApoE4 status; 3 in CDR-SB for the 0.5 group; 9 in MMSE; 26 in MoCA; 1 in Diagnosis; 8 in ApoE4 status; 3 in CDR-SB for the 0.8 group.

# Supplementary Figure 9.


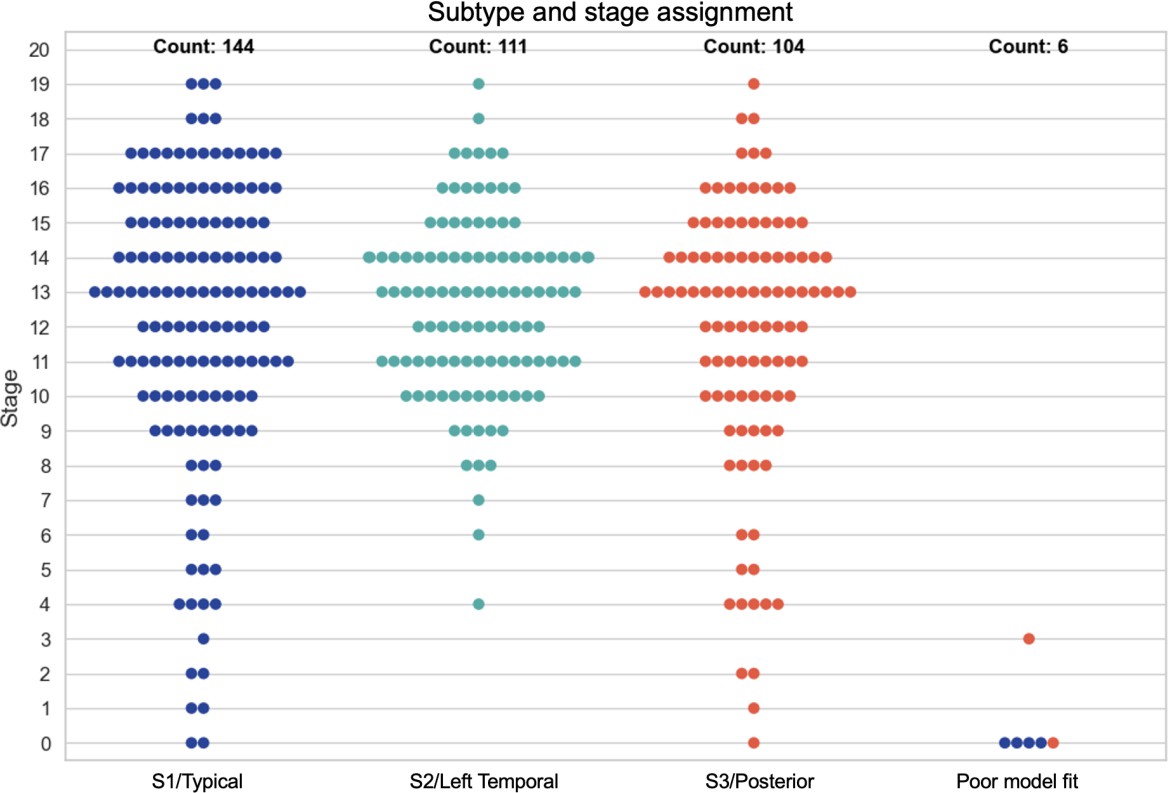


**Supplementary Figure 9. SuStaIn stage distribution across subtypes** and those with poor model fit (*n* = 6). Each data point represents one participant. The number of participants within each group is annotated above (Count). See supplementary table 3 for distribution statistics.

# Supplementary Table 8.

| **subtype** | **IQR [Q1, Q3]** | **mean** | **S.D.** | **kurtosis** | **skewness** |
| --- | --- | --- | --- | --- | --- |
| 1 | 5 [10, 15] | 12.09 | 4.21 | 0.47 | -0.88 |
| 2 | 3 [11, 14] | 12.56 | 2.6 | 0.37 | -0.27 |
| 3 | 4 [10, 14] | 11.78 | 3.93 | 0.67 | -0.98 |

**Supplementary Table 8. Stage distribution statistics for each subtype.**

# Supplementary Figure 10.


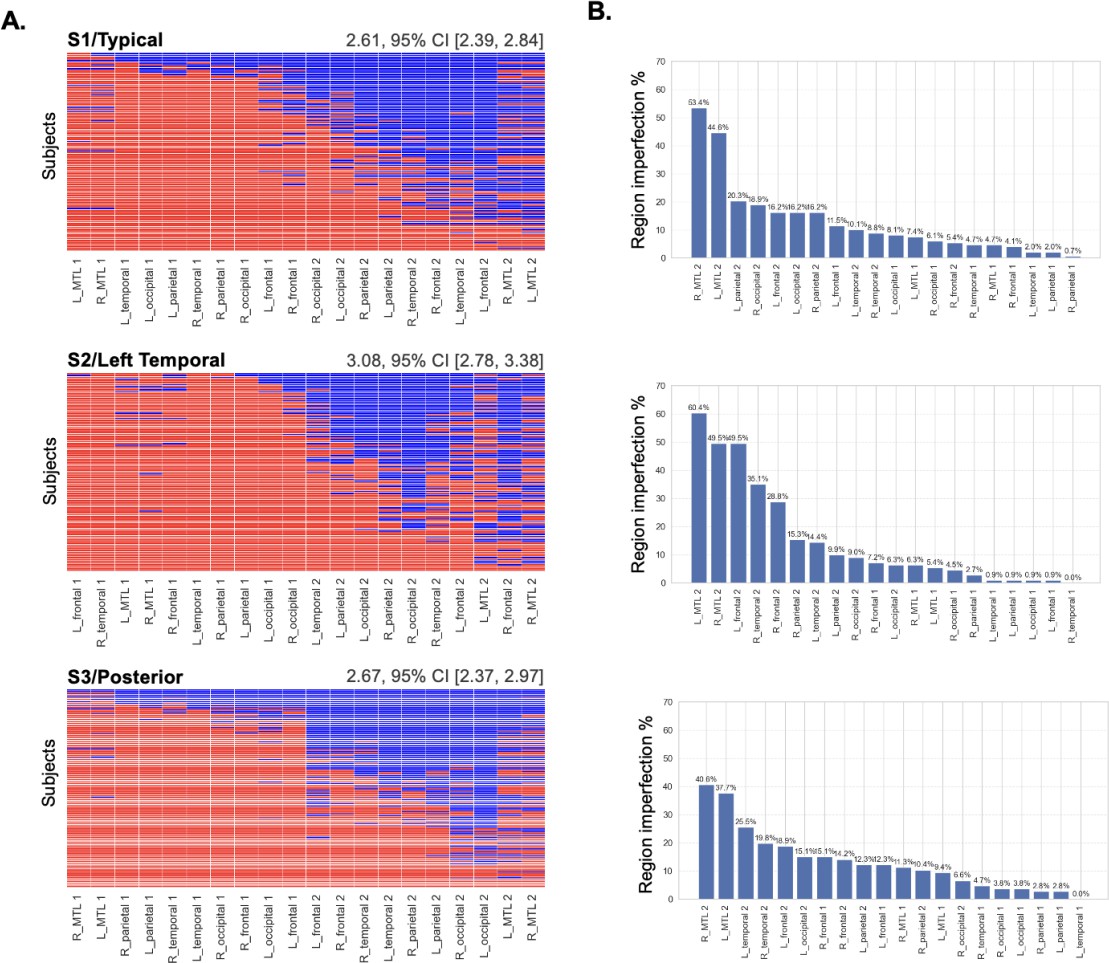


**Supplementary Figure 10. Event ordering and inconsistency. (A)** Event heatmaps showing the fit imperfection score for each participant across events within each SuStaIn subtype (S1/Typical: *n* = 144; S2/Left Temporal: *n* = 111; S3/Posterior: *n* = 104). Each row represents a participant, and each column represents a region-specific event in the subtype sequence. Red indicates that the participant’s regional [¹⁸F]Flortaucipir PET SUVR value has reached (i.e., fulfilled) the event-specific threshold, while blue indicates that it has not reached (i.e., unfulfilled) that threshold. The fit imperfection score quantifies the average number of unfulfilled events per participant relative to the model estimated subtype-specific 20-event sequence, with higher values reflecting greater deviation. Numbers above each heatmap indicate the mean fit imperfection score and its 95% confidence interval.
**(B)** Region-wise imperfection bar plots showing the percentage of participants with deviations from each event in the sequence, ordered from highest to lowest imperfection. These plots highlight consistent regional differences in event fulfillment, with certain ROIs (e.g., R_MTL2, L_MTL2) showing higher imperfection rates across subtypes.

# Supplementary Figure 11.


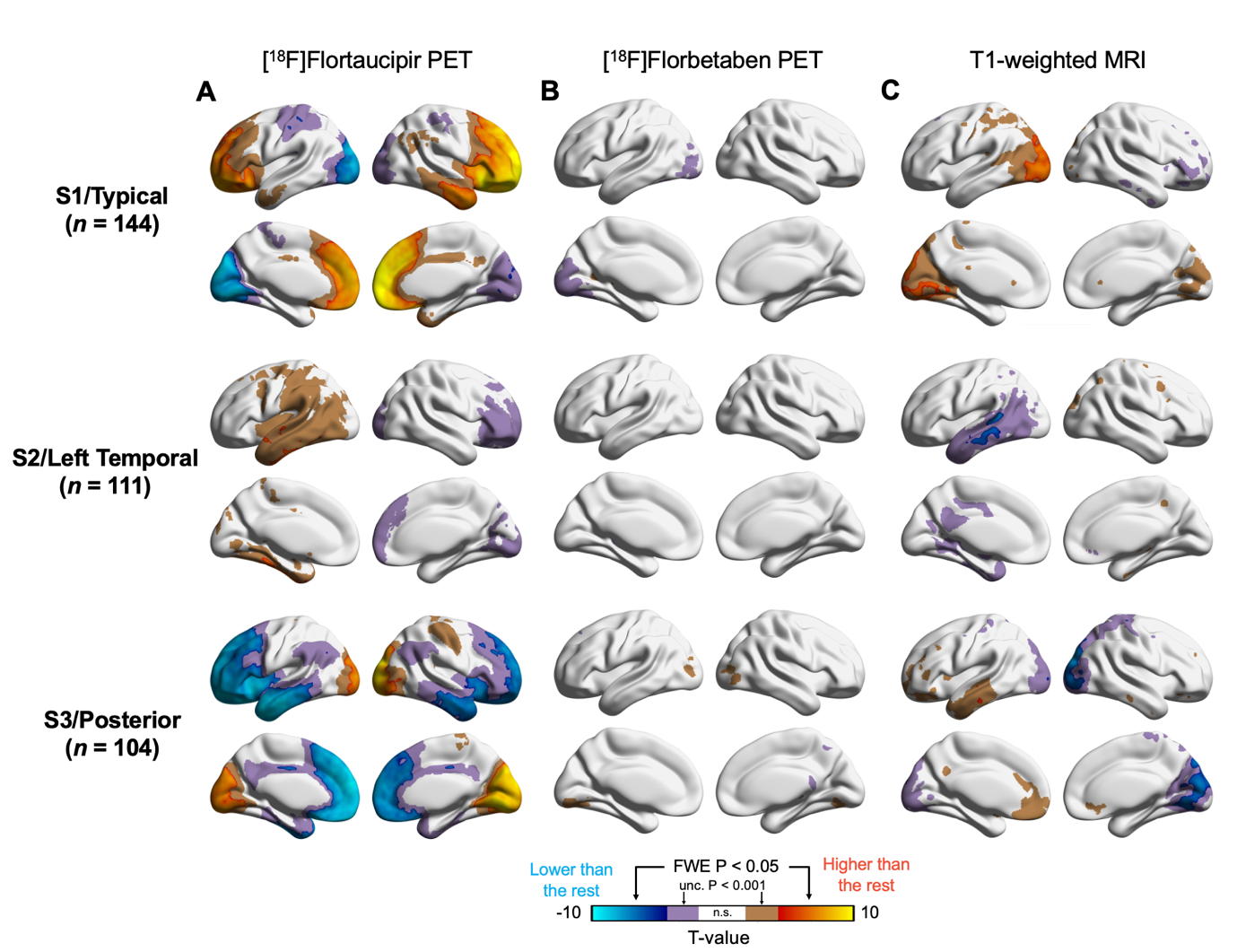


**Supplementary Figure 11. Voxel-wise baseline comparisons between each subtype (row) and the rest without covariate adjustment**. Each column corresponds to one data modality: **(A)** [^18^F]Flortaucipir PET, **(B)** [^18^F]Florbetaben PET, **(C)** T1-weighted MRI. Number of participants being assigned to each subtype is indicated on the left for each row. Voxel-wise statistical comparisons were performed for each subtype versus the rest of the subtypes using general linear models, where MRI models adjusted for Total Intracranial Volume. Colored areas indicate regions of statistically significant differences with a double threshold: uncorrected *P* < 0.001 (pale colors) and FWE corrected *P* < 0.05 (bright colors). n.s.: not significant.

# Supplementary Figure 12.


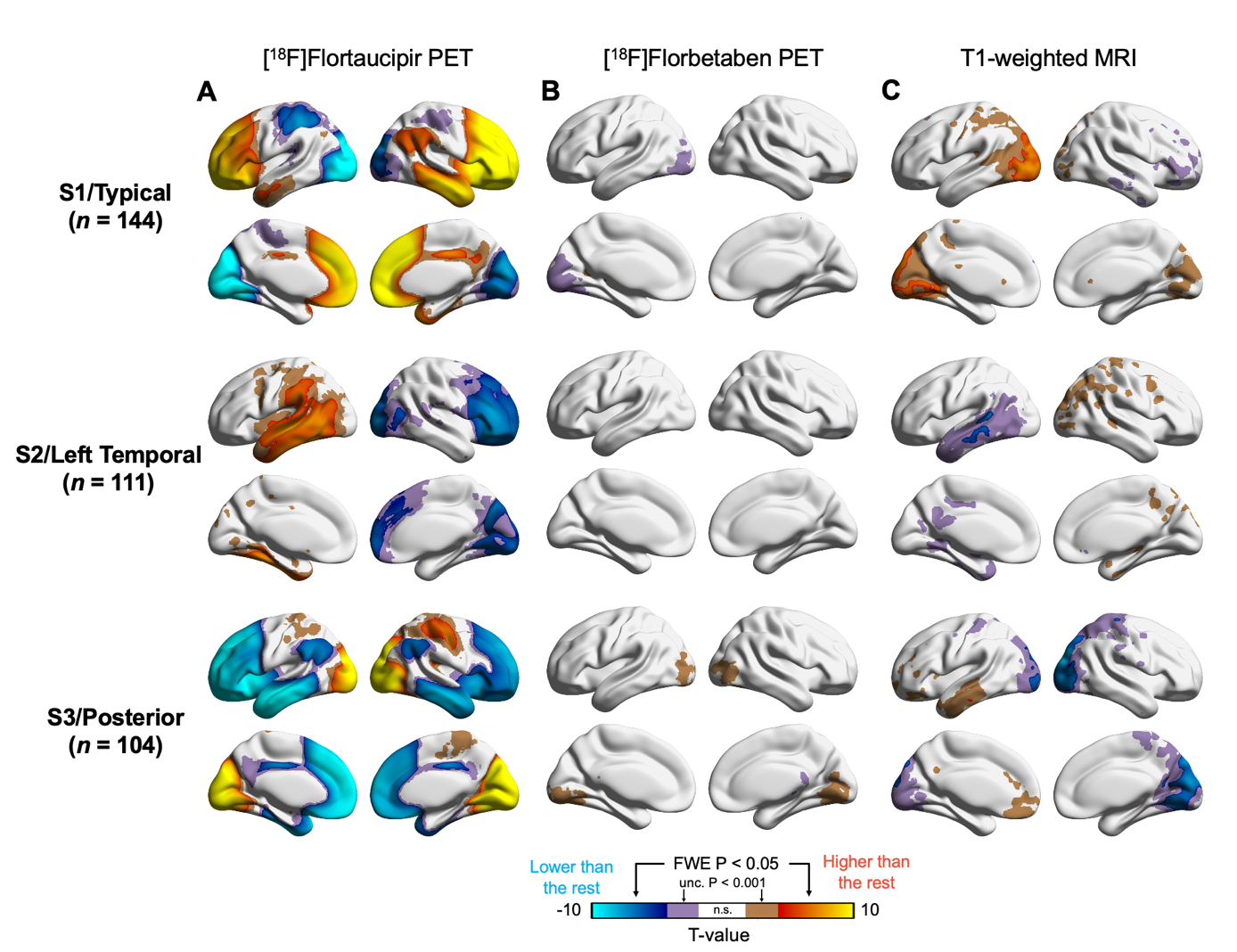


**Supplementary Figure 12. Voxel-wise baseline comparisons between each subtype (row) and the rest adjusting for baseline SuStaIn stage.** Number of participants being assigned to each subtype is indicated on the left for each row. Voxel-wise statistical comparisons were performed for each subtype versus the rest of the subtypes using general linear models adjusted for baseline SuStaIn stage where MRI models also adjusted for Total Intracranial Volume. Colored areas indicate regions of statistically significant differences with a double threshold: uncorrected *P* < 0.001 (pale colors) and FWE corrected *P* < 0.05 (bright colors). n.s.: not significant.

# Supplementary Table 9.

| **Variable** | **S1 vs. S2 *P*-value** | **S1 vs. S3 *P*-value** | **S2 vs. S3 *P*-value** |
| --- | --- | --- | --- |
| MoCA | 0.57 | 0.13 | **0.02** |
| Benson Figure Copy | 0.32 | **<0.001** | **0.05** |
| Line length | 0.98 | **0.03** | 0.07 |
| Line orientation | 0.99 | 0.07 | 0.08 |
| Multi-lingual Naming Test | **0.04** | 0.07 | **<0.001** |
| Semantic Fluency (Vegetable + Animals) | **0.03** | **0.04** | **<0.001** |
| Phonemic Fluency (L + F) | 0.33 | 0.22 | **0.01** |
| Craft Story 21 Delayed Recall | 0.24 | 0.41 | **0.02** |
| Trail Making Test Part A | 0.19 | **0.02** | 0.60 |
| Trail Making Test Part B | **0.05** | 0.94 | 0.11 |

**Supplementary Table 9.** *P*-values from post-hoc Tukey's tests for pairwise comparisons of baseline cognitive scores listed in Table 1.

# Supplementary Table 10.

|  |  | **Missing** | **Overall** | **Baseline only** | **Followed up** | ***P-*Value** |
| --- | --- | --- | --- | --- | --- | --- |
| n |  |  | 359 | 158 | 201 |  |
| **Demographics** |  |  |  |  |  |  |
| Years of Education |  | 0 | 15.6 (2.4) | 15.8 (2.5) | 15.5 (2.4) | 0.17 |
| Age |  | 0 | 59.1 (4.0) | 59.3 (3.8) | 58.9 (4.1) | 0.37 |
| Sex - Female |  | 0 | 198 (55.2) | 88 (55.7) | 110 (54.7) | 0.94 |
| ApoE4 Carrier |  | 9 | 195 (55.7) | 90 (59.6) | 105 (52.8) | 0.24 |
| Baseline [¹⁸F]Flortaucipir PET to data freeze time (month)* |  | 0 | 33.4 [15.9-53.2] | 24.8 [11.2-45.9] | 37.9 [23.6-54.9] | **<0.001** |
| **Neuroimaging** |  |  |  |  |  |  |
| [¹⁸F]Flortaucipir SUVR (all cortical ROIs) |  | 0 | 1.9 (0.4) | 1.9 (0.4) | 1.9 (0.4) | 0.71 |
| [¹⁸F]Flortaucipir SUVR (temporal Meta ROI) |  | 0 | 2.2 (0.5) | 2.2 (0.5) | 2.2 (0.5) | 0.95 |
| [¹⁸F]Florbetaben, Centiloid |  | 0 | 103.1 (27.9) | 100.2 (27.6) | 105.3 (27.9) | 0.09 |
| SuStaIn Stage |  | 0 | 12.1 (3.7) | 12.0 (3.9) | 12.3 (3.5) | 0.50 |
| **Clinical** |  |  |  |  |  |  |
| CDR-SB |  | 3 | 3.9 (2.0) | 4.1 (2.3) | 3.7 (1.7) | 0.05 |
| MMSE |  | 9 | 21.3 (5.4) | 20.8 (5.9) | 21.6 (4.9) | 0.17 |
| MoCA |  | 28 | 15.5 (6.2) | 15.4 (6.6) | 15.6 (5.8) | 0.83 |
| Diagnosis - Dementia |  | 1 | 267 (74.6) | 115 (73.2) | 152 (75.6) | 0.70 |
| Clinical Phenotype, n (%) | Amnestic CI | 0 | 290 (80.8) | 133 (84.2) | 157 (78.1) | 0.44 |
|  | Non-amnestic CI |  | 22 (6.1) | 8 (5.1) | 14 (7.0) |  |
|  | PCA |  | 24 (6.7) | 10 (6.3) | 14 (7.0) |  |
|  | PPA |  | 23 (6.4) | 7 (4.4) | 16 (8.0) |  |

**Supplementary Table 10.** Comparisons between participants with follow-up PET visits and participants with only baseline visits. *Median, [Interquartile Range]. Data freeze time = July 31^st^ 2024, *P*-value is from Wilcoxon rank-sum test.

# Supplementary Table 11.

|  |  | **Missing** | **Overall** | **Not changed** | **Changed** | ***P-*Value** |
| --- | --- | --- | --- | --- | --- | --- |
| *n* |  |  | 201 | 172 | 29 |  |
| **Demographics** |  |  |  |  |  |  |
| Years of Education |  | 0 | 15.5 (2.4) | 15.6 (2.4) | 14.8 (2.6) | 0.13 |
| Age |  | 0 | 58.9 (4.1) | 58.9 (4.2) | 59.5 (3.5) | 0.40 |
| Sex - Female |  |  | 110 (54.7) | 89 (51.7) | 21 (72.4) | 0.06 |
| ApoE4 Carrier |  | 2 | 105 (52.2) | 93 (54.1) | 12 (41.4) | 0.35 |
| **Neuroimaging** |  |  |  |  |  |  |
| [¹⁸F]Flortaucipir SUVR  (all cortical ROIs) |  | 0 | 1.9 (0.4) | 2.0 (0.4) | 1.8 (0.4) | 0.14 |
| [¹⁸F]Flortaucipir SUVR (temporal Meta ROI) |  | 0 | 2.2 (0.5) | 2.2 (0.5) | 2.1 (0.4) | 0.06 |
| [¹⁸F]Florbetaben, Centiloid |  | 0 | 105.3 (27.9) | 105.8 (27.5) | 102.4 (30.6) | 0.58 |
| SuStaIn Stage |  | 0 | 12.3 (3.5) | 12.5 (3.5) | 10.9 (3.3) | **0.02** |
| **Clinical** |  |  |  |  |  |  |
| CDR-SB |  | 0 | 3.7 (1.7) | 3.7 (1.6) | 3.6 (2.0) | 0.82 |
| MMSE |  | 2 | 21.6 (4.9) | 21.7 (4.8) | 21.3 (5.2) | 0.77 |
| MoCA |  | 11 | 15.6 (5.8) | 15.8 (5.7) | 14.4 (6.4) | 0.27 |
| Diagnosis - Dementia |  | 0 | 152 (75.6) | 129 (75.0) | 23 (79.3) | 0.79 |
| Clinical Phenotype, n (%) | Amnestic CI | 0 | 157 (78.1) | 136 (79.1) | 21 (72.4) | 0.22 |
|  | Non-amnestic CI |  | 14 (7.0) | 12 (7.0) | 2 (6.9) |  |
|  | PCA |  | 14 (7.0) | 13 (7.6) | 1 (3.4) |  |
|  | PPA |  | 16 (8.0) | 11 (6.4) | 5 (17.2) |  |

**Supplementary Table 11.** Comparisons between participants who changed subtype vs. those unchanged between baseline visit and last visit.

# Supplementary Table 12.

| **First vs Last** | | | |
| --- | --- | --- | --- |
| Subtype | Progressed | Regressed | Stable |
| 1 (*n* = 61) | 46 (75.7%) | 3 (5.4%) | 12 (18.9%) |
| 2 (*n* = 50) | 32 (64.1%) | 5 (9.4%) | 13 (26.6%) |
| 3 (*n* = 61) | 39 (64.6%) | 4 (6.2%) | 18 (29.2%) |
| Overall (*n* = 172) | 118 (68.5%) | 12 (6.9%) | 42 (24.6%) |
| **First vs Second** | | | |
| Subtype | Progressed | Regressed | Stable |
| 1 (*n* = 63) | 41 (65.6%) | 4 (6.2%) | 18 (28.1%) |
| 2 (*n* = 51) | 29 (56.9%) | 4 (7.8%) | 18 (35.3%) |
| 3 (*n* = 61) | 34 (55.7%) | 4 (6.6%) | 23 (37.7%) |
| Overall (*n* = 175) | 107 (61.4%) | 14 (7.9%) | 54 (30.7%) |

**Supplementary Table 12.** Summary of participants who progressed, regressed, or remained in their baseline SuStaIn stage between the first and the last, or between the first and the second visits, among those who remained in their baseline subtypes over time.

# Supplementary Figure 13.


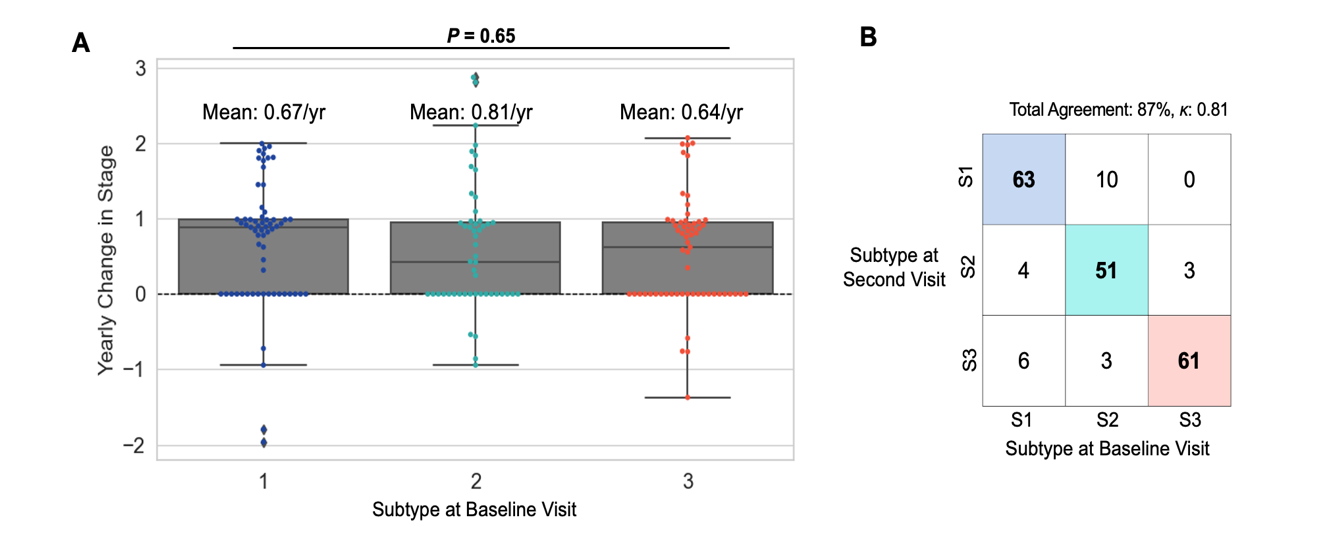


**Supplementary Figure 13. Longitudinal subtype stability and progression between first and second visits.** **(A)** Boxplots of yearly change in SuStaIn stage for each subtype, comparing first and second visits (S1: *n* = 63; S2: *n* = 51; S3: *n* = 61). Each data point represents one participant. Mean rate of change for each subtype is indicated above each box. One-way ANOVA test comparing yearly change in stage across subtypes showed no statistically significant differences (*F*(2, 172), *P* = 0.65). **(B)** Confusion matrix showing subtype stability across the first and second visits.

# Supplementary Figure 14.


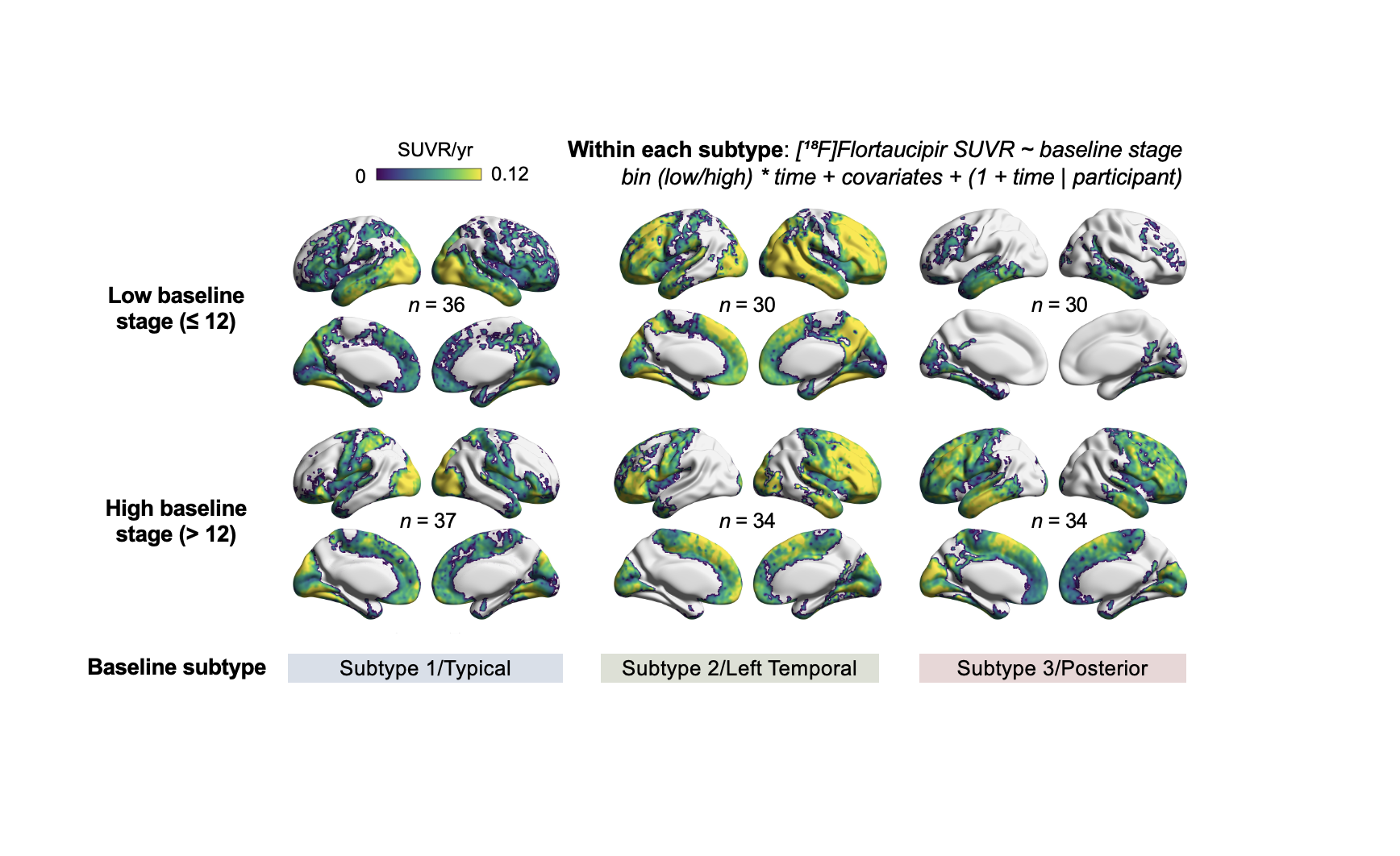


**Supplementary Figure 14. Baseline stage-stratified sensitivity analysis of longitudinal tau accumulation.** Within each baseline SuStaIn subtype, participants were stratified by baseline SuStaIn stage into low (≤12) and high (>12) stage bins, where 12 corresponds to the overall median. The maps showed voxel-wise annual rates of change in [¹⁸F]Flortaucipir SUVR estimated using linear mixed-effects models including time, stage bin, and their interaction, with participant-specific random intercepts and slopes, and adjusting for baseline Centiloids, age, sex, and years of education. The color scale indicates SUVR/year; *n* denotes the number of participants per group.

# Supplementary Figure 15.


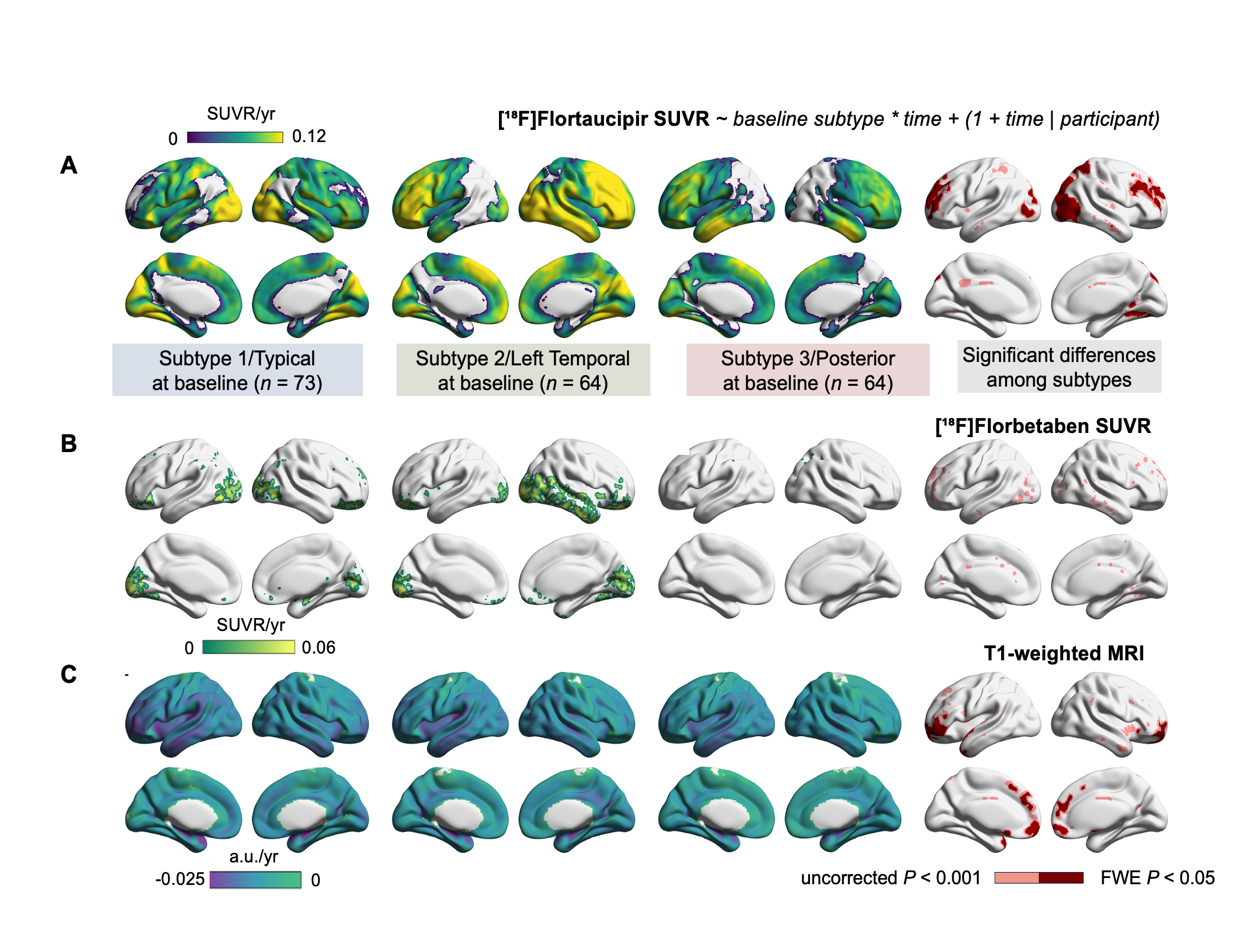


**Supplementary Figure 15. Longitudinal LME with no covariate adjustment.** Voxel-wise longitudinal analyses of **(A)** [¹⁸F]Flortaucipir PET, **(B)** [¹⁸F]Florbetaben PET, and **(C)** T1-weighted MRI, without covariate adjustment (aside from TIV for MRI). Each row represents a modality. The left three plots in each row display the estimated annualized rate of change, thresholded to show only regions with significant worsening over time within each group (FWE *P* < 0.05). The rightmost plot within each row highlights regions where rates of change were significantly different between subtypes with double thresholds as indicated by the red color bar. Voxel-wise LME models included fixed effects for baseline subtype, time from baseline, and their interaction, with the MRI models also adjusted for TIV. Random intercepts and slopes for time were included for each participant. a.u. = arbitrary unit.

# Supplementary Figure 16.


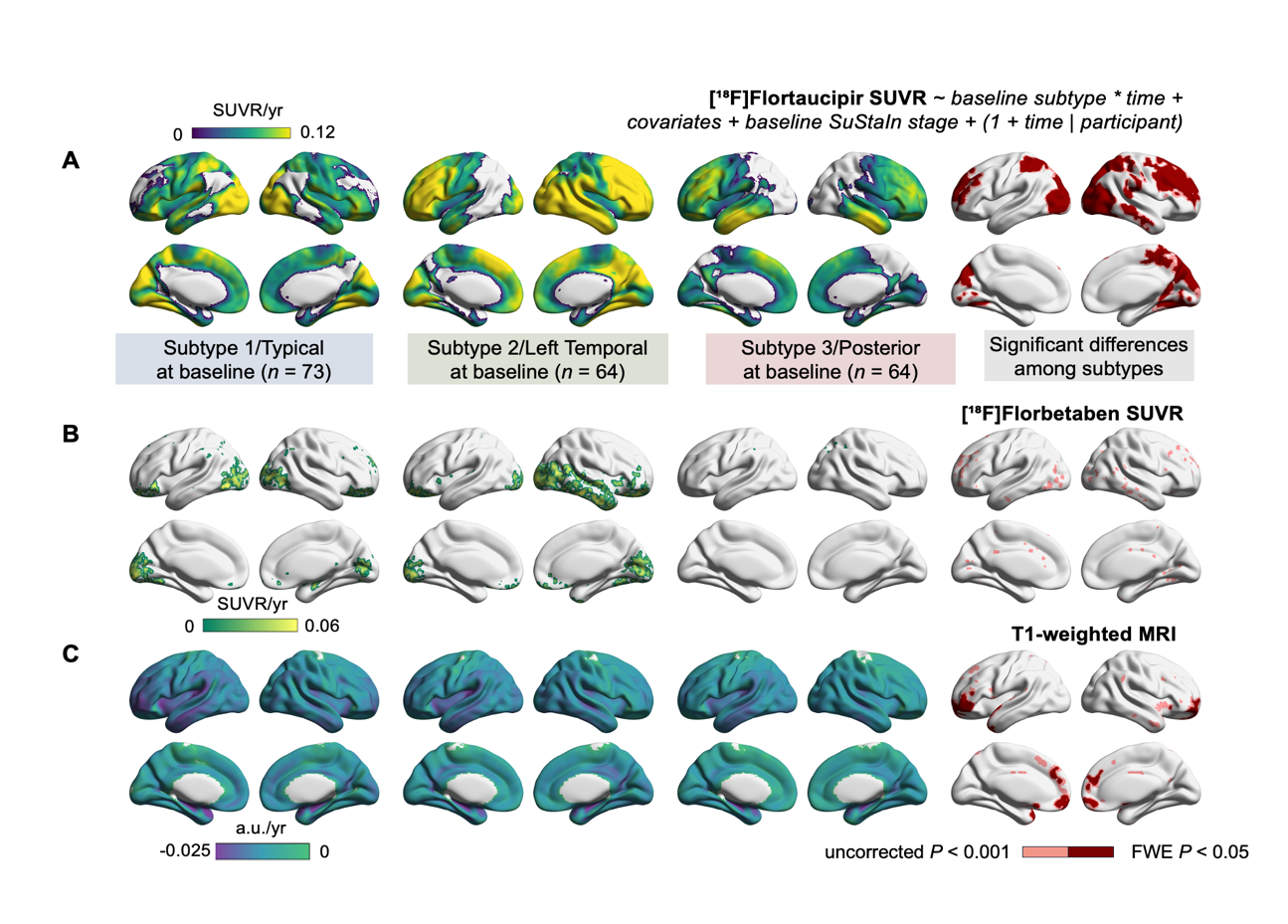


**Supplementary Figure 16. Longitudinal LME additionally adjusting for baseline SuStaIn stage.** Voxel-wise longitudinal analyses of **(A)** [¹⁸F]Flortaucipir PET, **(B)** [¹⁸F]Florbetaben PET, and **(C)** T1-weighted MRI. Each row represents a modality. The left three plots in each row display the estimated annualized rate of change, thresholded to show only regions with significant worsening over time within each group (FWE *P* < 0.05). The rightmost plot within each row highlights regions where rates of change were significantly different between subtypes with double thresholds as indicated by the red color bar. Voxel-wise LME models included fixed effects for baseline subtype, time from baseline, and their interaction, with covariate adjustment for baseline age, SuStaIn stage, sex, years of education, and Centiloid (excluded for [^18^F]Florbetaben PET models); MRI models also adjusted for TIV. Random intercepts and slopes for time were included for each participant. a.u. = arbitrary unit.

# Supplementary Figure 17.


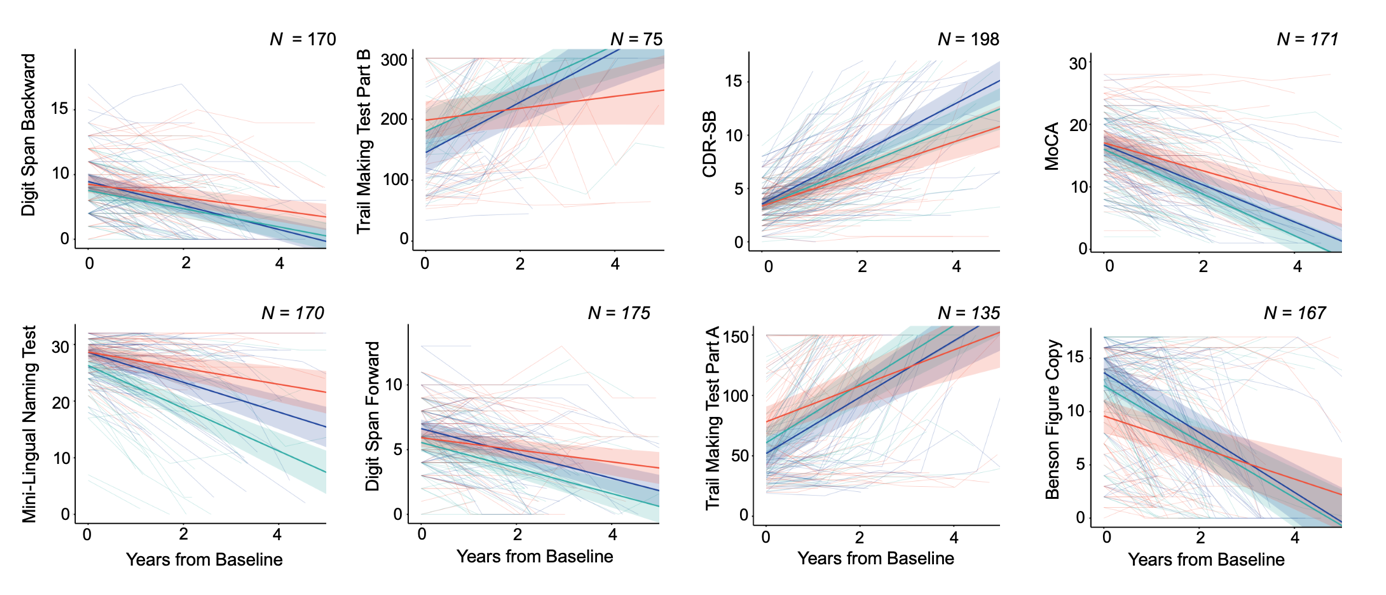


**Supplementary Figure 17. Longitudinal modeling of cognitive scores**, showing the individual-level trajectories (thin faded lines) and the model-predicted slopes with 95% confidence intervals (thick colored lines with shaded bands) estimated by LME models*. N* indicates the total number of participants contributing data for each panel. LME model specification, subtype-specific slope estimates, total number of test scores, and *P* values testing difference among slopes are reported in table 2.

# Supplementary Figure 18.

**
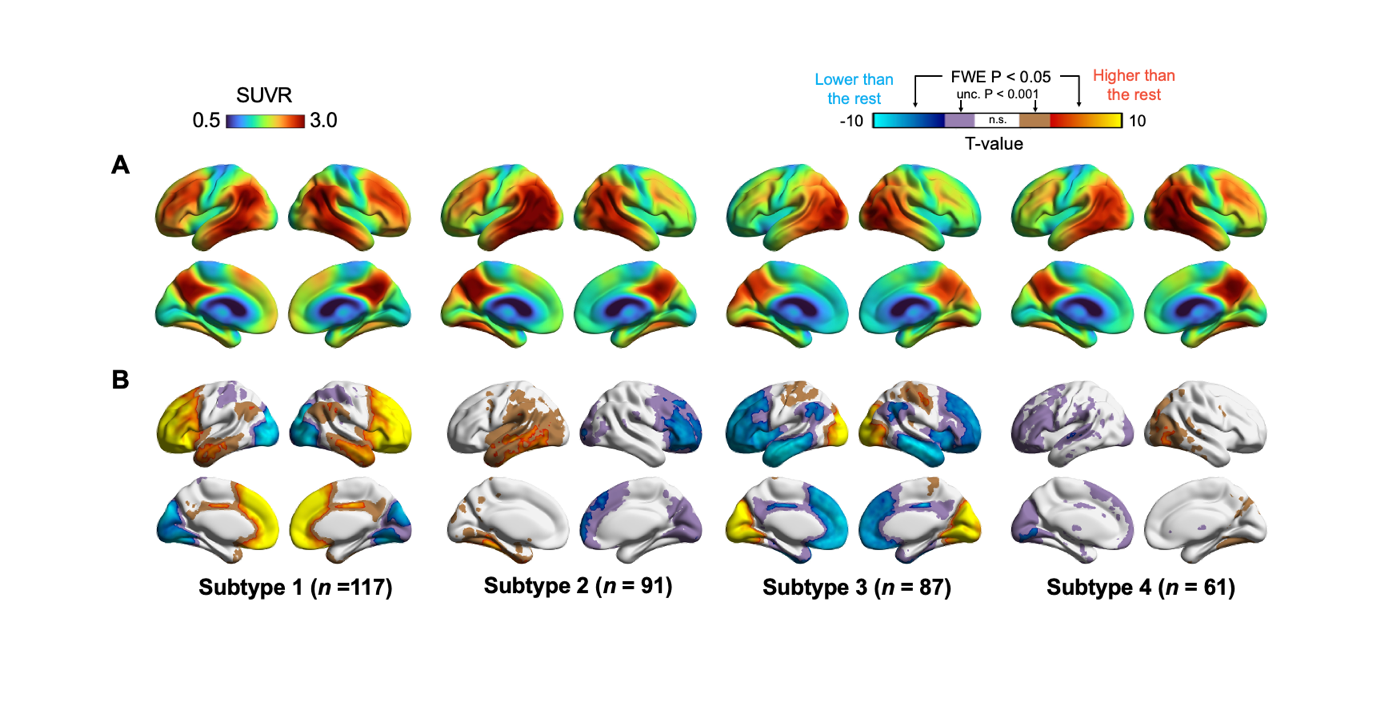
**

**Supplementary Figure 18. Voxel-wise baseline comparisons for the four-subtype solution.** Analyses adjusted for baseline SuStaIn stage and applying a 50% exclusion threshold for poor model fit. **(A)** Average [¹⁸F]Flortaucipir PET SUVR images. **(B)** Subtype-versus-rest comparisons using general linear models adjusted for baseline age, sex, years of education, SuStaIn stage, and Centiloid. Colored areas indicate regions of statistically significant differences with a double threshold: uncorrected *P* < 0.001 (pale colors) and FWE corrected *P* < 0.05 (bright colors). n.s.: not significant. Number of participants being assigned to each subtype is indicated below each column.

# Supplementary Table 13.

| **Variables** |  | **Missing** | **Total (*n* = 356)** | **S1 (*n* = 117)** | **S2 (*n* = 91)** | **S3 *(n* = 87)** | **S4 *(n* = 61)** | ***P*-Value** |
| --- | --- | --- | --- | --- | --- | --- | --- | --- |
| **Demographics** |  |  |  |  |  |  |  |  |
| Years of Education |  | 0 | 15.6 (2.4) | 15.5 (2.6) | 15.6 (2.4) | 15.9 (2.4) | 15.4 (2.4) | 0.64 |
| Age |  | 0 | 59.1 (4.0) | 58.6 (4.3) | 58.8 (4.0) | 59.6 (4.0) | 59.6 (3.3) | 0.22 |
| Sex – Female |  | 0 | 198 (55.6) | 67 (57.3) | 52 (57.1) | 47 (54.0) | 32 (52.5) | 0.91 |
| ApoE4 Carrier |  | 9 | 193 (54.2) | 60 (51.3) | 51 (56.0) | 55 (63.2) | 27 (44.3) | 0.21 |
| **Neuroimaging** |  |  |  |  |  |  |  |  |
| [¹⁸F]Florbetaben, Centiloid |  | 0 | 103.5 (27.7) | 104.8 (26.4) | 102.9 (25.6) | 100.0 (29.3) | 106.7 (30.5) | 0.48 |
| [¹⁸F]Flortaucipir SUVR (all cortical ROIs) |  | 0 | 1.9 (0.4) | 2.0 (0.5) | 2.0 (0.4) | 1.8 (0.4) | 1.9 (0.3) | 0.07 |
| [¹⁸F]Flortaucipir SUVR (temporal Meta ROI) |  | 0 | 2.2 (0.5) | 2.3 (0.6) | 2.4 (0.4) | 2.0 (0.4) | 2.3 (0.4) | **<0.001** |
| SuStaIn Stage |  | 0 | 12.3 (3.5) | 12.1 (4.2) | 12.6 (2.8) | 12.0 (3.8) | 12.6 (2.3) | 0.52 |
| **Clinical** |  |  |  |  |  |  |  |  |
| Diagnosis – Dementia |  | 1 | 268 (75.3) | 86 (73.5) | 69 (75.8) | 62 (71.3) | 51 (83.6) | 0.42 |
| Clinical Phenotype, n (%) | Amnestic CI | 0 | 288 (80.9) | 100 (85.5) | 73 (80.2) | 66 (75.9) | 49 (80.3) | **<0.001** |
|  | Non-amnestic CI |  | 21 (5.9) | 7 (6.0) | 5 (5.5) | 3 (3.4) | 6 (9.8) |  |
|  | PCA |  | 24 (6.7) | 2 (1.7) | 2 (2.2) | 15 (17.2) | 5 (8.2) |  |
|  | PPA |  | 23 (6.5) | 8 (6.8) | 11 (12.1) | 3 (3.4) | 1 (1.6) |  |
| MoCA |  | 28 | 15.4 (6.1) | 15.2 (6.5) | 14.5 (6.2) | 16.6 (6.0) | 15.6 (5.3) | 0.19 |
| CDR-SB |  | 3 | 3.9 (2.0) | 4.0 (2.3) | 3.8 (1.8) | 3.8 (1.8) | 4.2 (1.9) | 0.53 |
| MMSE |  | 9 | 21.2 (5.4) | 21.1 (5.9) | 20.3 (5.5) | 21.8 (4.9) | 21.9 (4.4) | 0.18 |

**Supplementary Table 13. Sensitivity analysis of the four-subtype solution**. Summary of demographic, clinical, and cognitive variables for each of the four subtypes and group comparisons. See Table 1 for details.
